# Multiomic Reference Map of Endothelial Mechanosensitive Pathways Under Athero‐ and Erosion‐Prone Flow

**DOI:** 10.1161/JAHA.125.048290

**Published:** 2026-06-23

**Authors:** Giulio Vidotto, Sara Luzzi, Jonathan D. Humphries, Robert Beal, David G. McVey, Christopher P. Nelson, Thomas R. Webb, Martin J. Humphries, Graham R. Smith, Stephen J. White

**Affiliations:** ^1^ Bioinformatics Support Unit, Faculty of Medical Sciences Newcastle University Newcastle upon Tyne United Kingdom; ^2^ Biosciences Institute, Faculty of Medical Sciences Newcastle University Newcastle upon Tyne United Kingdom; ^3^ Department of Life Sciences Manchester Metropolitan University Manchester United Kingdom; ^4^ Leicester British Heart Foundation Centre of Research Excellence & Division of Cardiovascular Sciences University of Leicester and National Institute for Health Research Leicester Biomedical Research Centre Leicester United Kingdom; ^5^ Wellcome Centre for Cell‐Matrix Research, Faculty of Biology, Medicine & Health University of Manchester Manchester United Kingdom

**Keywords:** endothelial cells, gene expression, plaque erosion, shear stress, Basic Science Research, Cell Biology/Structural Biology, Endothelium/Vascular Type/Nitric Oxide, Physiology, Vascular Biology

## Abstract

**Background:**

Atherosclerosis develops at arterial sites exposed to disturbed flow, whereas plaque rupture and plaque erosion predominantly occur in regions subjected to elevated flow. The impact of elevated flow on regulation of endothelial gene expression is less well studied; therefore, we undertook a comprehensive analysis of primary human coronary artery endothelial cell gene expression under elevated flow, comparing it to gene expression induced by normal physiological and oscillatory flow.

**Methods:**

Analysis of human coronary artery endothelial cell messenger RNA, microRNA and protein expression cultured under oscillatory shear stress, physiological laminar shear stress, and elevated shear stress (ESS) for 72 hours. Identification of changes in RNA isoform expression and proximity of flow‐responsive genes to established coronary artery disease risk loci were also performed.

**Results:**

A total of 2175 shear‐regulated genes were identified, with 665 uniquely responsive to ESS. Both ESS and oscillatory shear stress induced significant changes in RNA isoform selection, predicted to affect 848 and 580 genes respectively. Signaling pathways regulating coronary artery disease pathogenesis including Hippo, TGFβ/BMP (transforming growth factor beta/bone morphogenetic protein), and IRF (interferon regulatory factor), showed altered RNA isoform selection, which may influence plaque development and plaque erosion. A total of 65% of linkage disequilibrium‐filtered coronary artery disease‐associated genetic variants contained at least 1 oscillatory shear stress or ESS‐regulated gene within 250 kb. Proteomic analysis identified 289 proteins differentially expressed under oscillatory shear stress and 171 under ESS, with notable discordance between messenger RNA and protein changes observed in 28.7% (oscillatory shear stress versus laminar shear stress) and 16.6% (ESS versus laminar shear stress) genes. Additionally, 40 shear‐responsive microRNAs were identified.

**Conclusions:**

Elevated flow elicits a distinct gene expression program in human coronary artery endothelial cells, modulating pathways central to coronary artery disease pathogenesis.

**Nonstandard Abbreviations and Acronyms**
DEA: differential expression analysis
DEGs: differentially expressed genes
ESS: elevated shear stress
HCAEC: human coronary artery endothelial cell
LSS: physiological laminar shear stress
miRs: microRNAs
OSS: oscillatory shear stress

**Research Perspective**

**What Is New?**

- Plaque erosion predominantly occurs on the upstream surface of the plaque, where the endothelium is exposed to elevated flow, which we show within this study to evoke a significant and largely unique regulation of the transcriptome, miRNome and proteome within primary human coronary artery endothelial cells.
- Identify shear stress as a significant regulator of alternative splicing, with elevated flow causing the greatest shift in alternative transcript selection, and identify 135 differentially expressed genes in oscillatory flow in proximity to coronary artery disease risk loci and 101 in elevated flow.

**What Question Should Be Addressed Next?**

- Study of the response of the coronary endothelium directly in patients to understand how the risk factors involved in plaque erosion change endothelial function, and creation of physiological 3‐dimensional arterial models replicating in vivo observations to study the precipitating factors involved in plaque erosion.

Coronary artery disease (CAD) and its acute and chronic clinical manifestations remain one of the greatest contributors of worldwide morbidity and mortality. The hemodynamic environment modifies many aspects of endothelial behavior, with low average shear stress and disturbed “athero‐prone” flow promoting plaque development and progression.^1^, ^2^, ^3^, ^4^, ^5^ By contrast, plaque disruption most frequently occurs in regions of the plaque exposed to elevated flow. This is true for both plaque rupture and plaque erosion, which represent the most frequent contributors to atherothrombotic causes of myocardial infarction and predominantly occur on the upstream surface and area of minimal lumen area of stenotic plaques where the endothelium encounters elevated flow.^6^, ^7^, ^8^

Although the endothelial response to disturbed or oscillatory flow has received significant attention, the response of the endothelium to elevated flow, where acute coronary syndromes most frequently occur, has not been studied in depth.^9^ To address this shortfall, we present an in‐depth multiomic assessment of the response of primary human coronary artery endothelial cells (HCAECs) to elevated flow, with comparison to normal physiological laminar flow and contrast this to the response to oscillatory flow. Compared with normal physiological flow, elevated and oscillatory flow both significantly regulate gene expression, splicing, microRNA (miR) expression and protein expression, although in distinct and largely nonoverlapping manner. Additional analysis revealed a significant enrichment of oscillatory or elevated flow‐regulated genes within 250 kb of genomic loci known to carry risk for development of CAD identified by genome‐wide association studies.^10^, ^11^

## METHODS

### Data Availability

The combined RNAseq and miRseq data sets are available at GSE300941. Proteomics data are available via ProteomeXchange with identifier PXD075178. In addition, a complete description of the bioinformatic methodology, including scripts for each step including merging miR databases and machine learning are available at https://github.com/GiulioVidotto/Influence_of_miRs_in_regulating_the_response_of_endothelial_cells_to_different_flow_environments

### HCAEC Culture and Generation of RNA and Protein Samples for Analysis

Confluent monolayers of 3 independent batches of male HCAECs purchased from Promocell were exposed to oscillatory shear stress (OSS)±0.5 Pa 1 Hz, normal physiological laminar shear stress (LSS; 1.5 Pa) and elevated laminar shear stress (ESS; 7.5 Pa) for 72 hours using our established parallel plate flow chambers as previously described.^9^, ^12^, ^13^, ^14^ In brief, passage 3 and 4 HCAECs were seeded at high density onto gelatin‐coated slides and cultured for 72 hours to ensure confluence and allow stable cell–cell junctions to form and extracellular matrix deposition to occur. Subsequently these HCAECs were cultured under flow for 72 hours before lysis for either RNA or protein purification. The ability to run >25 flow chambers at a time allowed the protein and RNA samples for each individual donor to be prepared in a single experiment, allowing direct comparison across the different omic analyses for each HCAEC donor. Each donor RNA or protein sample represents one replicate in each analysis, therefore there was n=3 RNA or protein samples analyzed. Further detail is supplied in the Data Set S1. As the HCAECs were purchased from Promocell and have replicated outside the body, they are not bound by the human tissue act and are exempt from the need for ethical review.

### Statistical Analysis

Optimized statistical analysis, including adjustments for multiple testing for RNAseq, miRseq, splicing analysis, proteomics, CAD‐associated loci analysis, and machine learning models are detailed in Data S1, along with a full description of the methodology. In brief, for RNAseq, normalization and differential gene expression tests were performed in DESeq2 (v1.42.1) with confirmation of normalization through *P* value analysis. Splicing was assessed within rMATS, using the paired statistical model with a cutoff (−‐cstat) of 0.05. Results calculated by counting both the junction and the exon reads were filtered to only include differential splicing events with a *P* value ≤0.01 and predicted differential PSI of 8%. Proteomics were analyzed within Proteome Discoverer (v2.5; ThermoFisher Scientific) with adjusted *P* values calculated using the Benjamini–Hochberg method. Determination of differentially expressed gene (DEG) association with CAD risk loci was assessed through permutation testing.

## RESULTS

### Pathological Flow Induces Distinct Gene Expression Changes in Endothelial Cells

**Figure 1:**
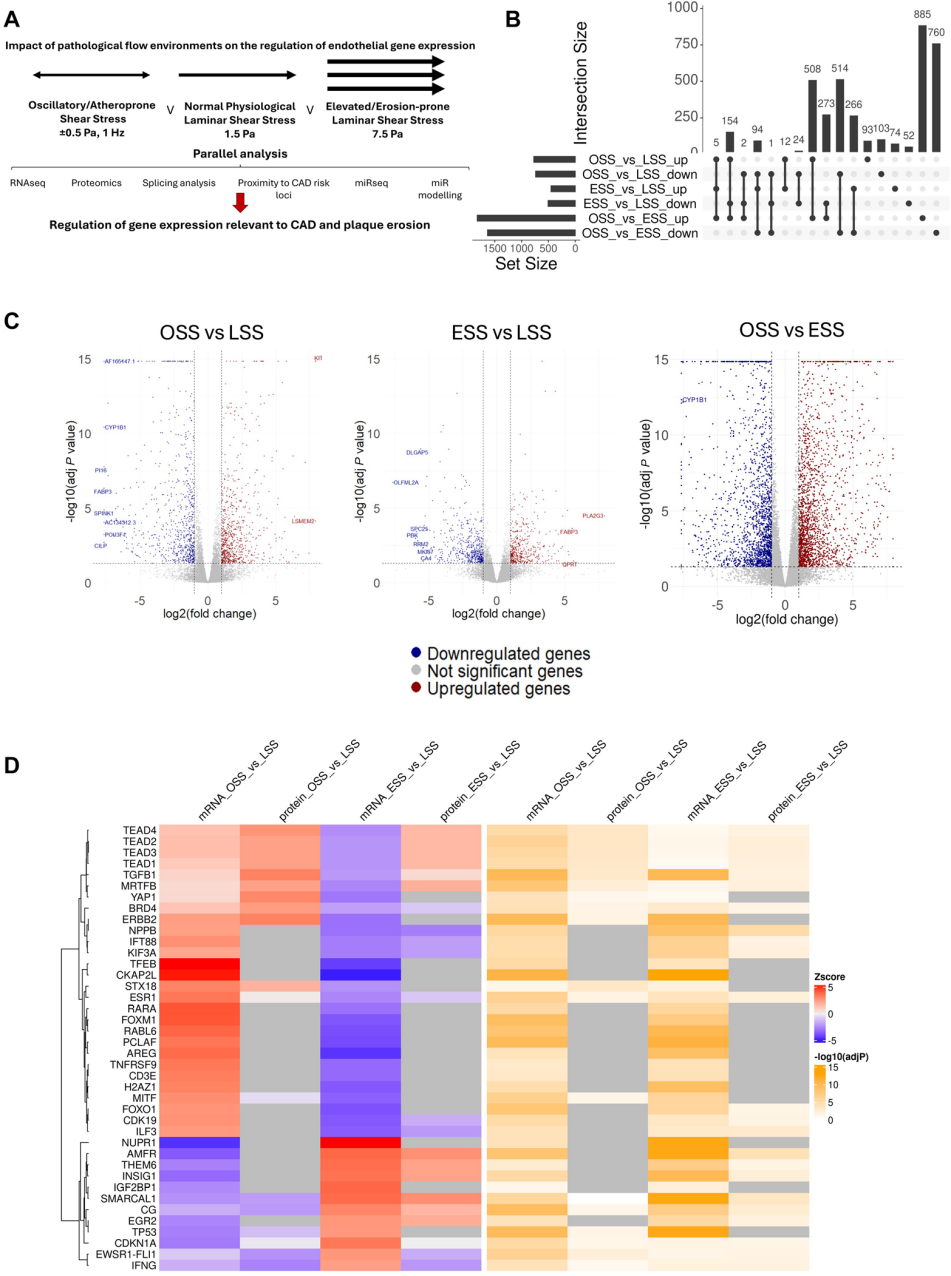
Analysis of the impact of oscillatory and elevated flow on mRNA expression. **A**, Graphical overview of the analysis pipeline. **B**, Upset plot of the differentially expressed genes. This plot shows the overlap of differentially expressed genes between OSS, physiological LSS, and ESS. **C**, Volcano plot visualization of the differential expression analysis for the mRNA‐seq in the OSS vs LSS, ESS vs LSS and OSS vs ESS conditions. The *x* axis shows the log_2_FC values, and the *y* axis represents the −log_10_ of the adjusted *P* values. Genes with log_2_FC≤−1 and adjusted *P* value ≤0.05 are labeled as downregulated (blue), and genes with log_2_FC≥1 and adjusted *P* value ≤0.05 are labeled as upregulated (red). Genes that do not meet these criteria are considered not significant (gray). The volcano plot for the mRNA‐seq in the OSS vs LSS condition is on the left and the one for the mRNA‐seq in the ESS vs LSS condition is on the right. **D**, Heatmap of the top upstream regulators in the OSS vs LSS and ESS vs LSS conditions. This plot shows the top 40 upstream regulators identified using ingenuity pathway analysis at the transcriptomics and proteomics levels. The *Z* score represents the predicted activation (red) or inhibition (blue) of the upstream regulators, while significance is shown as negative log_10_ of the adjusted *P* values. Gray boxes identify missing data. CAD indicates coronary artery disease; ESS, elevated shear stress; log_2_FC, log_2_ of the fold change; LSS, laminar shear stress; mRNA, messenger RNA; and OSS, oscillatory shear stress.

We performed multiomic analyses using HCAECs from 3 independent male donors. The samples for all the omics on an individual donor were collected at the same time, allowing direct comparisons of data generated across the different omic analyses (Figure 1A, principal component analysis plot Figure S1). RNAseq demonstrated that compared with physiologically laminar flow of 1.5 Pa (LSS), oscillatory flow ±0.5 Pa 1Hz (OSS) uniquely regulated 1218 DEGs >2 fold, *P*adj<0.05, whereas elevated laminar flow 7.5Pa (ESS) regulated 675 DEGs >2 fold, *P*adj<0.05. A total of 302 DEGs showed >2‐fold regulation by both OSS and ESS (*P*adj<0.05, Figure 1B, 1C). Significantly regulated genes are listed in Data Set S1. Examination of the expression of arterial markers (*SEMA3G*, *NOTCH4*, *GJA5*—relatively highly expressed, *GJA4*, variable between donors), vein markers (*ACKR1*, *VCAM1*‐ both expressed at low levels), capillary markers (*CA4*, *RGCC*, *HEY1*, *F2RL3*—moderate expression) summarized in,^15^ would suggest the HCAECs used in this study generally maintained an arterial or capillary‐like phenotype in culture (Table S1). To highlight the difference between the response of HCAEC to culture under OSS and ESS, a direct comparison was performed identifying 3481 genes (>2 fold, *P*adj<0.05, Data Set S1) were altered between these 2 flow environments underlining the uniqueness of these 2 “activated” gene expression programs.

A combined analysis of shear regulated genes at the messenger RNA (mRNA) and protein level (described in detail next) revealed a dominant role for TEADs (transcriptional enhanced associate domain), YAP (Yes‐associated protein), and TGFβ (transforming growth factor beta), aligning to previously reported shear regulatory programs (Figure 1D, genes related to each pathway are listed in Data Set S1). In addition, MRTFB (myocardin‐related transcription factor B) a cotranscriptional activator of *SRF* (serum response factor) shows a consistent upregulation in OSS and downregulation under ESS. *TP53* (p53) and *CDKN1A* (p21) gene programs change in a way consistent with the established control of cell cycle by physiological laminar flow.

Of note, there were a number of hypervariable genes, with a >8‐fold mean increase or decrease expression in either OSS or ESS that failed to reach significance due to variation between donors (*P*adj>0.05, Data Set S1), particularly highlighting genes that constitute a donor‐specific response to flow. Further investigation of these hypervariable genes in much larger data sets may provide mechanistic insight into patient‐specific CAD risk.

### Pathway Prediction Based on mRNA Changes

**Figure 2:**
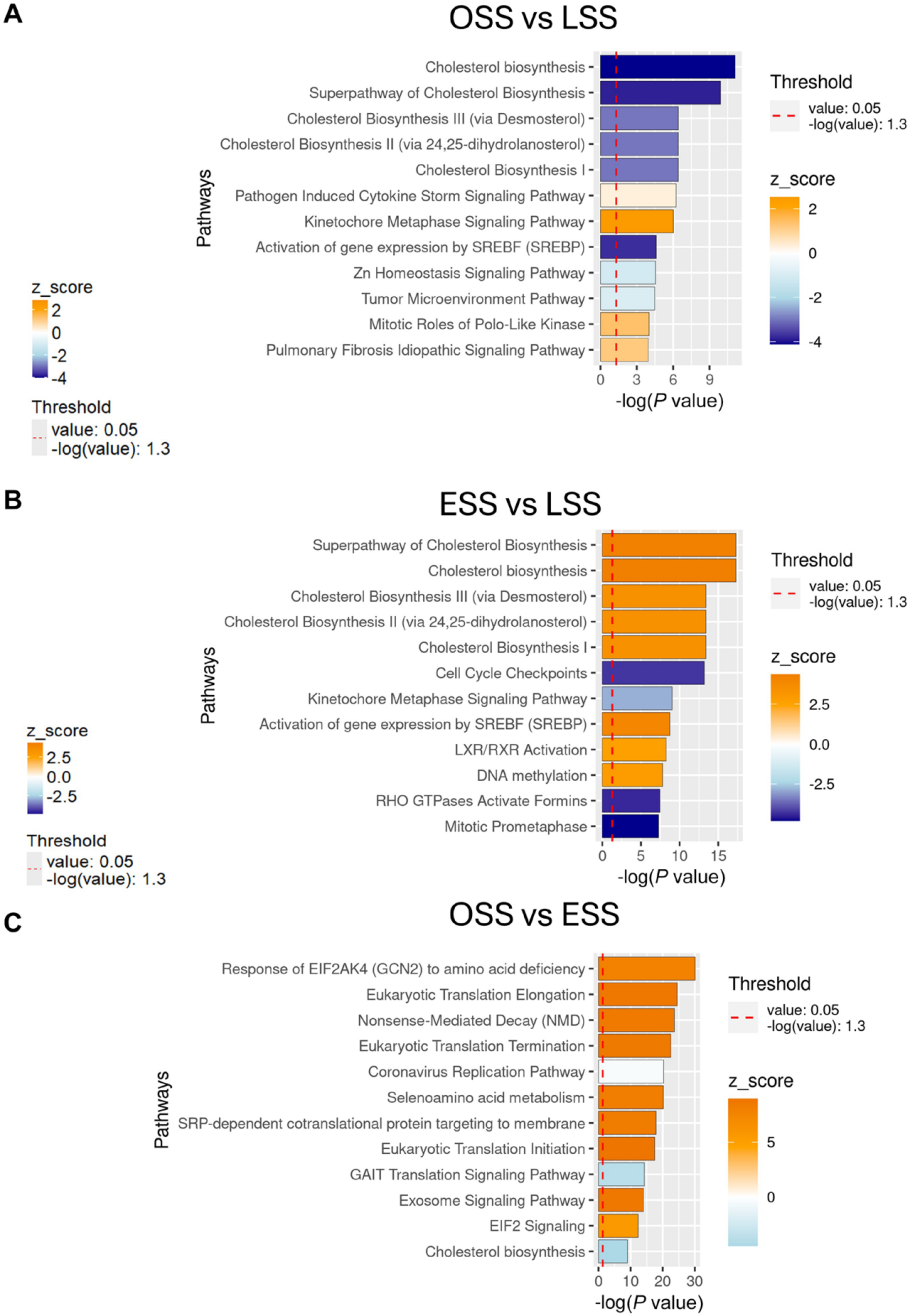
Pathway analysis of differentially regulated genes. **A,** **B**, and **C**, Horizontal bar charts of the canonical pathways at the transcriptomic level in OSS, physiological LSS, and ESS. The plots show the most enriched pathways at the transcriptomic level for the OSS vs LSS (**A**), ESS vs LSS (**B**) and OSS vs ESS (**C**) comparisons. On the *y* axis are shown the names of the enriched pathways, ordered based on the significance and the *x* axis displays the negative log_10_ of the *P* values of overlap. Each bar is color‐coded according to the *Z* score, with orange and blue bars in the bar chart indicating predicted pathway activation or predicted inhibition, respectively. ESS indicates elevated shear stress; LSS, physiological laminar shear stress; and OSS, oscillatory shear stress.

Alteration in activation and suppression of signaling pathways, based on mRNA changes provided an interesting insight into the function of OSS and ESS DEGs (Figure 2A through 2C, with pathway‐related genes listed in Data Set S2, S3). Repetition within overlapping pathways were removed through simplifying the pathways in R using binary distance between the gene sets associated with each pathway and hierarchical clustering via the hclust algorithm (Figures S4 and S5) with the most significant pathway in each cluster presented in Figure 2. All pathways are listed in Figures S6 and S7 along with networks showing related pathways (Figures S8 and S9). Both shear environments were dominated by the regulation of cholesterol synthesis, predicted to be low in OSS and high in ESS. This was consistent with the observed downregulation of *INSIG1*‐dependent gene expression in OSS and upregulation in ESS (Figure 1D). Genes controlling cell cycle are again consistent with the known interaction between laminar flow and cell proliferation. The direct comparison between OSS and ESS (Figure 2C) reinforced the change in cholesterol synthesis pathways (elaborated in the network analysis in Figure S10) but highlighted additional differences in the control of transcription and translation, at least inferring that the global gene expression machinery is elevated under OSS, likely required for increased proliferation resulting from the activation of *CDK1* and *CDK2* (Figure S10).

### Protein Expression and Pathway Analysis

**Figure 3:**
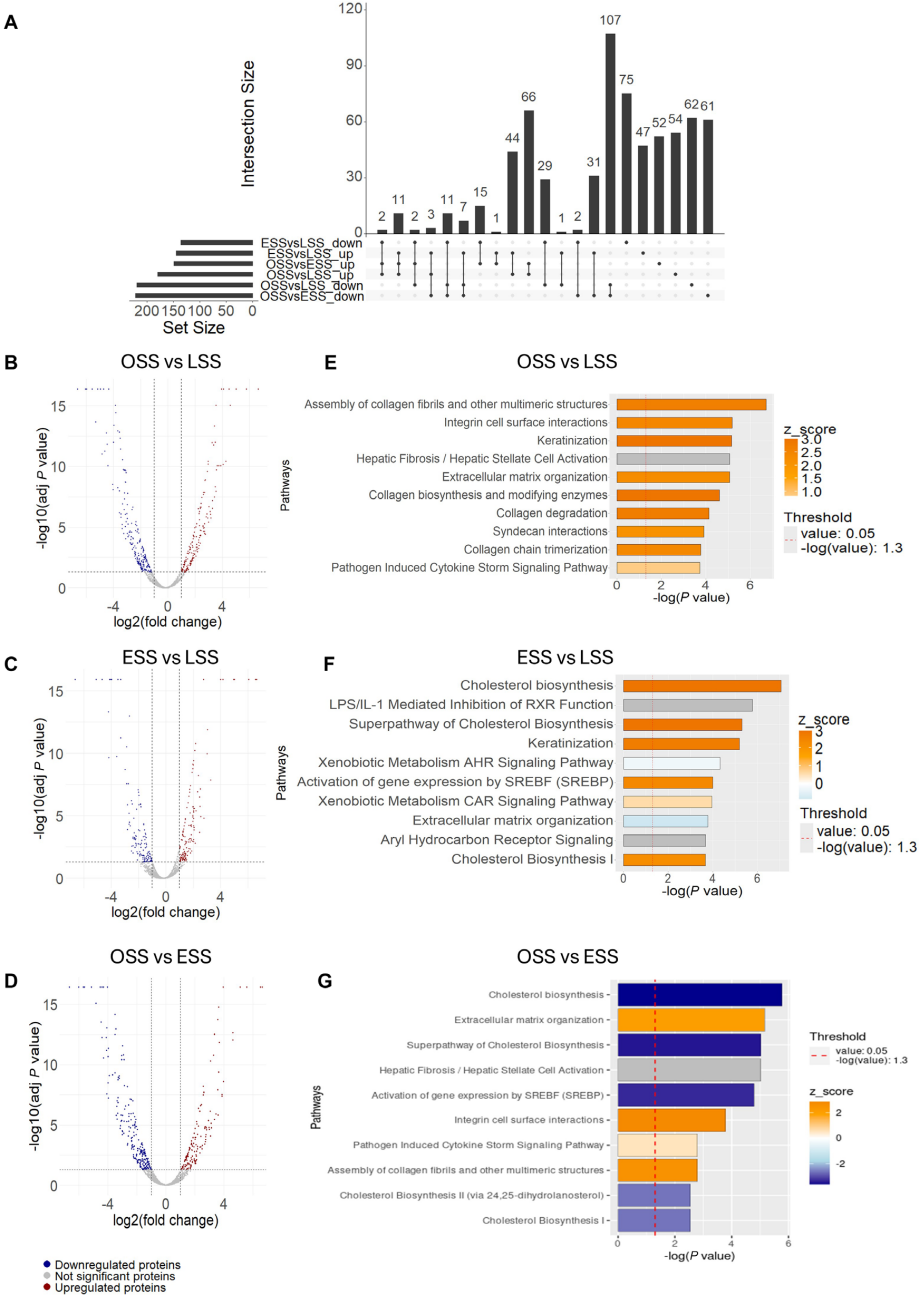
Analysis of the impact of oscillatory and elevated flow on protein expression. **A**, Upset plot of the differentially expressed proteins. This plot shows the overlap of differentially expressed proteins between ESS, LSS and OSS . Each section represents upregulated (Up) and downregulated (Down) proteins that are either unique to 1 condition or shared between both. **B**, **C and D**, Volcano plots of the result of DEA for the proteins in the OSS vs LSS, ESS vs LSS and OSS vs ESS conditions. The *x* axis shows the log_2_FC values, and the *y* axis represents the −log_10_ of the adjusted *P* values. Genes with log_2_FC≤−1 and adjusted *P* value ≤0.05 are labeled as downregulated (blue), and genes with log_2_FC≥1 and adjusted *P* value ≤0.05 are labeled as upregulated (red). Proteins that do not meet these criteria are considered not significant (gray). **B**, DEA results for proteomics in the OSS vs LSS contrast. **C**, DEA results for proteomics in the ESS vs LSS contrast, **D**, DEA results for the OSS vs ESS contrast. **E**, **F** and **G** Horizontal bar charts of the canonical pathways at the proteomics level in the OSS vs LSS, ESS vs LSS and OSS vs ESS conditions. On the *y* axis are shown the names of the enriched pathways, ordered based on the significance and the *x* axis displays the negative log_10_ of the *P* values of overlap. Each bar is color‐coded according to the *Z* score, with orange and blue bars in the bar chart indicating predicted pathway activation or predicted inhibition, respectively. **E**, Canonical pathways identified at the proteomics level for the OSS vs LSS condition. **F**, Canonical pathways identified at the proteomics level for the ESS vs LSS condition, **G,** Canonical pathways identified at the proteomics level for the OSS vs ESS condition. DEA indicates differential expression analysis; ESS, elevated shear stress; log_2_FC, log_2_ of the fold change; LSS, laminar shear stress; and OSS, oscillatory shear stress.

High stringency liquid chromatography tandem mass spectrometry analysis of HCAEC proteome identified a total of 597 shear‐regulated proteins, compared with 2175 genes by RNAseq (>2 fold, *P*adj<0.05) (Figure 3A through 3D and Data Set S1). As with the transcriptomic analysis, there were substantial numbers of distinctly regulated proteins under both OSS and ESS.

Many pathways related to extracellular matrix organization and collagen biosynthesis, were significantly regulated at the level of protein expression (Figure 3E through 3G). As this analysis was performed on confluent HCAEC monolayers cultured under flow for 72 hours allowing the accumulation of extracellular matrix, the inclusion of the extracellular matrix within the protein sample provides additional focus on the extracellular compartment allowing the contribution to extracellular matrix to more clearly observed. Consistent with the RNAseq results, cholesterol biosynthesis pathways were activated under ESS, emphasized in the direct comparison between OSS and ESS. These results add new information in addition to the analysis of gene expression at the RNA level, with informatic analysis providing insight into the upstream regulators and causal networks (Data Set S3).

### Oscillatory and Elevated Shear Stress Have Distinct Impact on Production of Alternative RNA Isoforms

**Figure 4:**
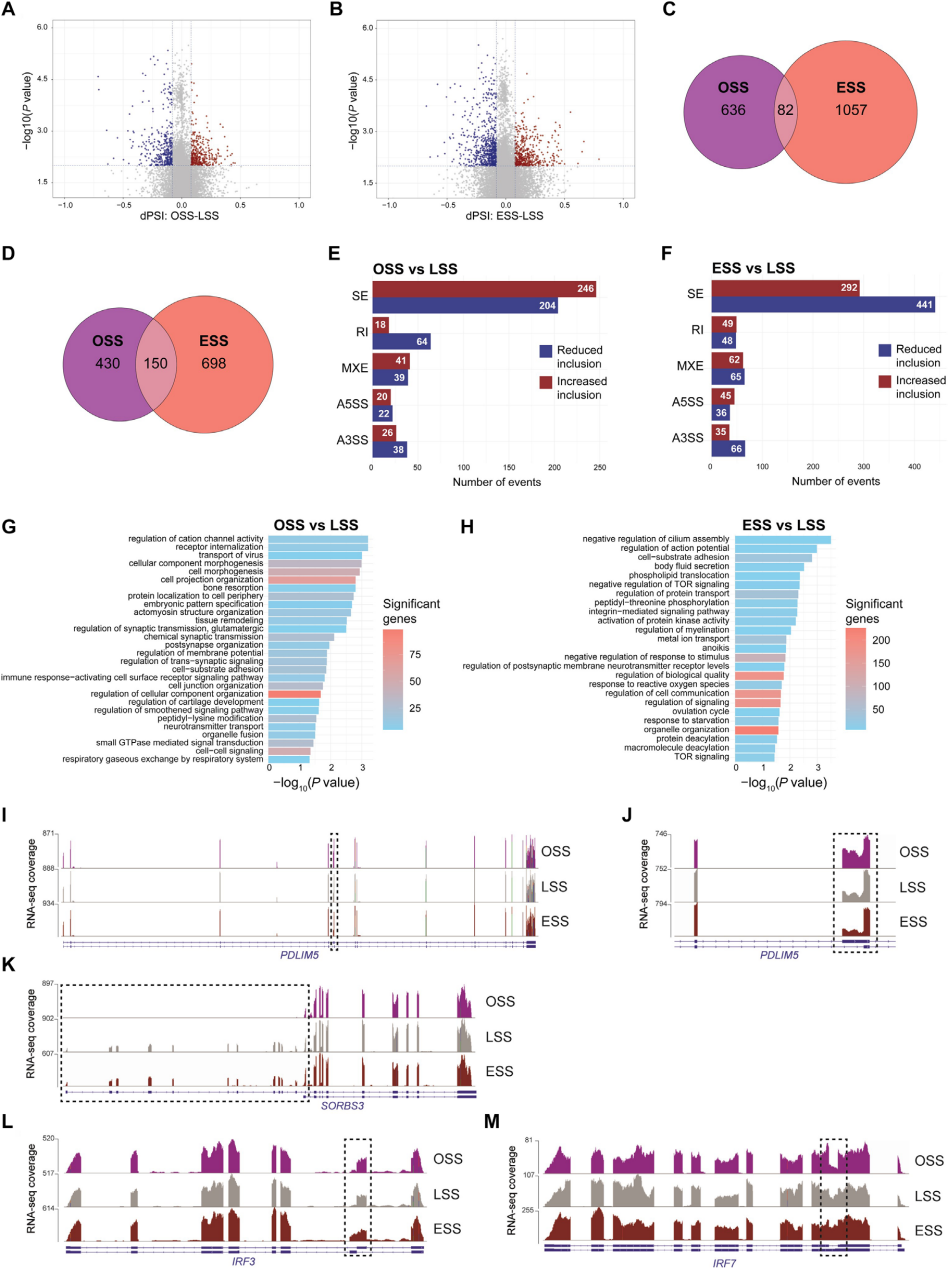
The impact of OSS, physiological LSS, and ESS on RNA transcript selection. **A**, Volcano plot shows alternative splicing events within protein‐coding genes identified in OSS compared with LSS as calculated using rMATS v.4.1.2. Blue and red dots represent splicing events with significantly reduced (dPSI≤−0.08 and *P* value <0.01) or significantly increased inclusion (dPSI ≥0.08 and *P* value <0.01) in OSS compared with LSS. **B**, Analysis as in **A**, but for alternative splicing events identified in ESS compared with LSS. **C** and **D**. Venn diagrams show common differential alternative splicing events (**C**) or common genes undergoing differential alternative splicing (**D**) in both OSS and ESS compared with LSS. **E**, Bar plot illustrates types of significant alternative splicing events identified in OSS compared with LSS. For each, the number of events showing significantly reduced (blue bars) and significantly increased (red bars) inclusion levels are indicated. **F**, Analysis as in **E**, but for alternative splicing events identified in ESS compared with LSS. **G**, Bar plot shows gene ontology analysis of genes that undergo alternative splicing in OSS compared with LSS. Colors indicate the number of significant genes belonging to the respective biological process. **H**, Analysis as in **G**, but for alternative splicing events identified in ESS compared with LSS. **I‐M**, Snapshot of RNA‐seq merged tracks from the 3 batches of HCAECs subjected to OSS, LSS, and ESS from the Integrative Genomics Viewer genome browser showing significant differential alternative splicing events over *PDLIM5*, *SORBS3*, *IRF3*, and *IRF7*. A3SS indicates alternative 3′ splice site; A5SS, alternative 5′ splice site; dPSI, differential percentage splicing inclusion; ESS, elevated shear stress; HCAEC, human coronary artery endothelial cell; LSS, laminar shear stress; MXE, mutually exclusive exons; OSS, oscillatory shear stress; RI, intron retention; and SE, exon skipping/inclusion.

To further understand the impact of shear stress on gene expression, we sought to gain insights into alternative RNA processing. Alternative RNA splicing plays a key role in cardiovascular function and has been associated to cardiovascular disease including atherosclerosis.^16^, ^17^, ^18^ However, the impact of elevated flow on alternative splicing has not been previously studied. Therefore, we performed differential splicing analyses using rMATS and identified a significant impact of shear stress on regulation of transcript selection. N=3 has sufficient power for this analysis.^19^ Compared with LSS, OSS and ESS resulted in 727 or 1158 alternative splicing events respectively (Figure 4A through 4D). The effect of OSS and ESS on alternative splicing is largely distinct, with only 150 genes (26% of OSS versus LSS and 18% of ESS versus LSS) being differentially spliced in both OSS and ESS, and only 82 splicing events (11% of OSS versus LSS and 7% of ESS versus LSS) appear differentially regulated in both OSS and ESS compared with LSS. The majority of predicted splicing changes involve exon skipping/inclusion, with ESS leading to a particularly high number of exon skipping events; however, OSS resulted in markedly reduced intron retention (Figure 4E, 4F; gene lists Data Set S1). This analysis spotlights that significant modulation of gene expression in response to shear stress occurs through regulation of the abundance of alternative RNA isoforms, in addition to overall transcriptional control. Gene Ontology analyses revealed a number of common themes including regulation of cell surface receptors, cell–cell junction components, and membrane and cytoskeletal organization. Of potential importance for plaque erosion, elevated flow modulated splicing of genes involved in cell substrate adhesion, integrin‐mediated signaling, response to reactive oxygen species and TOR (target of rapamycin) signaling (Figure 4G, 4H, and Data Set S1).

Of relevance to CAD, the YAP/TAZ (transcriptional co‐activator with PDZ‐binding motif) signaling regulators *PDLIM5* and *SORBS3* displayed OSS‐specific alternative splicing patterns (Figure 4I through IK). Specifically, OSS but not ESS led to increased inclusion of an alternative 3′ splice sites within *PDLIM5* RNA predicted to introduce 109 amino acids within the disordered domain, compared with the protein isoform expressed in LSS. Furthermore, OSS caused a dramatic switch in *SORBS3* isoform expression leading to a shorter RNA transcript expressing a protein isoform that lacks the Sorbin Homology domain, which enables interactions with cytoskeletal proteins like vinculin.^20^ On the other hand, ESS but not OSS also caused significant changes in alternative splicing patterns of YAP/TAZ activators, in particular *TEAD1* and *PARD3* (Data Set S1), resulting in the expression of a shorter TEAD1 protein isoform and a reduced hinge between the PARD3 (partitioning defective 3) PDZ domains. Interestingly, ESS and OSS also led to changes in the interferon regulators *IRF3* and *IRF7* respectively (Figure 4L, 4M), both affecting selection of the first methionine during protein translation. ESS caused increased selection of a mutually exclusive exon with predicted production of a shorter *IRF3* (interferon regulatory factor) protein isoform lacking the N‐terminal domain, which includes the nuclear localization signal. OSS enhanced recognition of an exitron (internal region of an exon spliced like an intron), which would result in selection of a different N‐terminal domain. Production of these alternative isoforms, alongside observed changes in IRF regulators *IRAK4* and *STING1* (Data Set S1), may alter sensitivity of IRF signaling, implicated to play a role in plaque erosion.^21^

### Expression and Regulation of Putative CAD Risk Loci Genes in HCAEC

**Figure 5:**
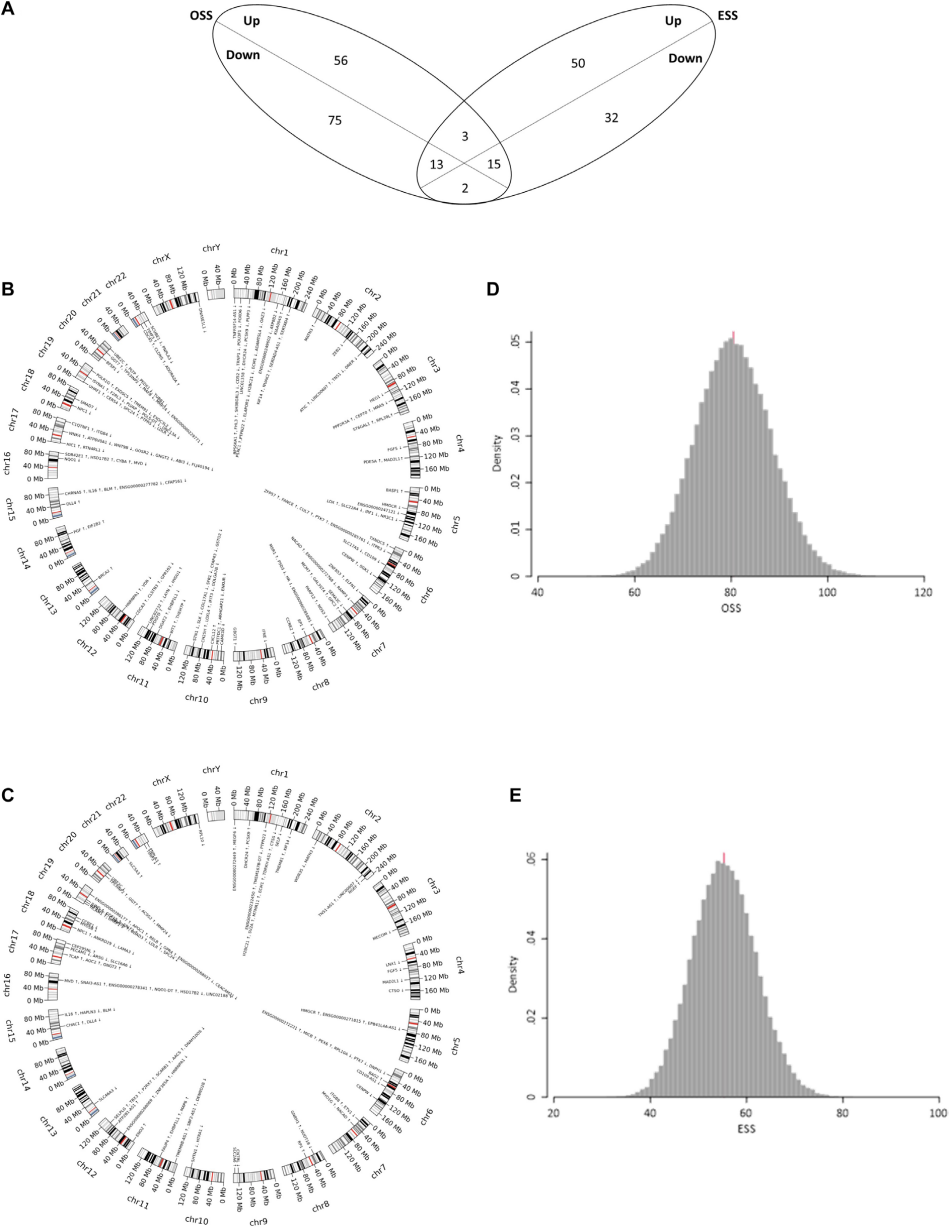
Proximity of OSS and ESS differentially regulated genes to CAD risk loci. **A**, Venn diagram of the OSS and ESS differentially expressed genes within 250 kb of a CAD‐associated variant. This plot shows the number of differentially expressed genes under OSS and ESS conditions whose gene start site is located within 250 kb of a CAD‐associated variant. Each section indicates the shear stress type (OSS or ESS) and the direction of the gene expression change under the indicated condition compared with physiological LSS. **B**, Circos plot showing the approximate location of OSS differentially expressed genes within 250 kb of a CAD‐associated variant. Circos plots showing the approximate locations of all OSS differentially expressed genes (compared with laminar flow) within 250 kb of a CAD‐associated variants. Up arrows indicate genes whose expression was increased under OSS vs LSS, and down arrows indicate genes whose expression was decreased under OSS vs LSS. Locations are approximate to ensure legibility of all labels. **C**, Circos plot showing the approximate location of ESS differentially expressed genes within 250 kb of a CAD‐associated variant. Circos plots showing the approximate locations of all ESS differentially expressed genes (compared with laminar flow) within 250 kb of a CAD‐associated variants. Up arrows indicate genes whose expression was increased under ESS vs LSS, and down arrows indicate genes whose expression was decreased under ESS vs LSS. Locations are approximate to ensure legibility of all labels. **D**, Histogram showing the permutation distribution of OSS. The results from 100 000 permutations identifying differentially expressed genes (compared with laminar flow) near randomly selected genetic variants. The mean is shown in red with the maximum for OSS random variants being 114, compared with 135 found for CAD variants. **E**, Histogram showing the permutation distribution of ESS. The results from 100 000 permutations identifying differentially expressed genes (compared with laminar flow) near randomly selected genetic variants. The mean is shown in red with the maximum for ESS random variants being 87, compared with 101 found for CAD variants. CAD indicates coronary artery disease; ESS, elevated shear stress; LSS, laminar shear stress; and OSS, oscillatory shear stress.

Genome‐wide association studies has identified numerous genetic loci containing single nucletide variants that associate with variable risk of CAD. To determine whether the DEGs identified in the OSS and ESS conditions were located near to CAD risk loci, a list of linkage disequilibrium‐filtered variants from 2 recent CAD multipopulation meta‐analyses was compiled^10^, ^11^ (375 variants in total with MAF>0.005) and calculated the distance between DEG start sites and CAD variants. This analysis considered 1495 OSS DEGs and 954 ESS DEGs from chr 1–22 and chr X. A total of 135 OSS DEGs and 101 ESS DEGs were located within 250 kb of a CAD risk variant; 36% of the CAD‐associated variants had at least 1 OSS DEG within 250 kb, and 26.93% were within 250 kb of at least 1 of the ESS DEGs. A summary of the DEGs within 250 kb of the CAD variants is shown in Figure 5A, genes and single‐nucleotide variants (SNVs) listed in Data Set S1. Circos plots indicating the approximate locations of all OSS and ESS DEGs within 250 kb of CAD‐associated variants are shown in Figure 4B and 4C respectively.

An enrichment analysis was performed to investigate whether the association between the 375 CAD lead SNVs and shear‐regulated DEGs might occur by chance. Repeated analysis of 375 similar, but randomly selected SNVs 100 000 times identified the probability of observing the association with CAD risk loci by random chance was extremely small (*P*<1×10^−6^ for both OSS and ESS, Figure 5D, 5E). This highlights a potential role for shear regulated DEGs in participating in the genetically identified pathogenesis of CAD.

### The Hemodynamic Environment Modifies the Expression of miRs

**Figure 6:**
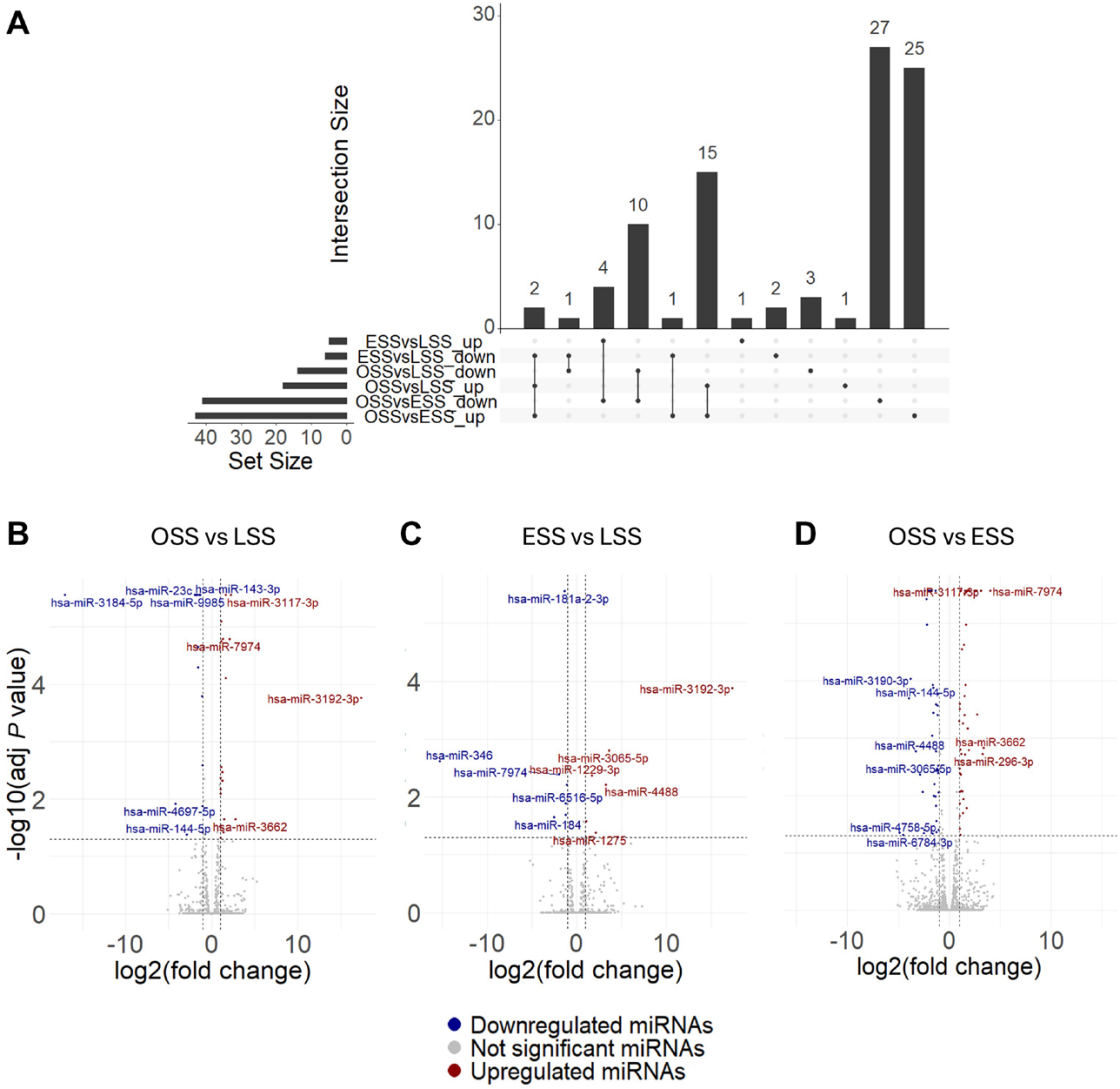
Analysis of the impact of oscillatory and elevated flow on miRNA expression. **A**, Upset plot of the differentially expressed miRNAs. This plot shows the overlap of differentially expressed miRNAs between ESS and OSS conditions. Each section represents upregulated (Up) and downregulated (Down) miRNAs that are either unique to 1 condition or shared between both. **B‐D**. Volcano plots of the result of DEA for the miRNA‐seq in the OSS vs physiological LSS, ESS vs LSS, and OSS v ESS conditions. The *x* axis shows the log_2_FC values, and the *y* axis represents the −log_10_ of the adjusted *P* values. Genes with log_2_FC≤−1 and adjusted *P* value ≤0.05 are labeled as downregulated (blue), and genes with log_2_FC≥1 and adjusted *P* value ≤0.05 are labeled as upregulated (red). Genes that do not meet these criteria are considered not significant (gray). **B**, DEA results for miRNAs in the OSS vs LSS contrast. **C**, DEA results for miRNAs in the ESS vs LSS contrast. **D**, DEA results for miRNAs in the OSS vs ESS contrast. DEA indicates differential expression analysis; ESS, elevated shear stress; log_2_FC, log_2_ of the fold change; LSS, laminar shear stress; miRNA, microRNA; and OSS, oscillatory shear stress.

Sequencing of the miRNome of HCAECs identified that HCAECs express a total of 1113 miR. In contrast to changes in mRNA expression, comparatively few miRs were regulated by shear stress (>2‐fold, *P*adj<0.05, >10 normalized average counts summed across all conditions, Figure 6A through 6C) listed Data Set S1, again showing limited overlap between the OSS and ESS‐regulated miRs. OSS versus LSS had the greatest effect on miR expression, regulating a total of 32 miR, with ESS versus LSS regulating only 11 miR >2 fold. The direct comparison between OSS and ESS again showed the greatest number of regulated miRs with 84 mir regulated >2 fold. Despite the relatively small number of significantly regulated miRs, their predicted regulation of genes showing discordance between their mRNA and protein expression was deemed significant (see next section) indicating an important role in regulating endothelial gene expression in response to shear stress.

### Comparison Between mRNA and Protein Expression Identified Genes With Discordant Gene Expression

We aligned the expression of significantly detected proteins with their cognate mRNAs to examine concordance between mRNA and protein expression. We found that 2122 genes (49.9% of all detected genes) were not regulated at either mRNA or protein expression by any flow environment. A total of 145 flow regulated genes demonstrated a concordant change in both mRNA and protein expression by shear stress. By contrast, 28.7% (OSS versus LSS) and 16.6% (ESS versus LSS) of flow‐regulated genes demonstrate discordant regulation between RNA and protein expression (listed in Data Set S1, Figures S11 and S12, top 5 genes in each group presented in Table S2).

To examine whether targeted protein degradation was potentially responsible for discordant gene expression, we examined if these discordantly regulated genes were enriched for KFERQ‐like motifs using KFERQ finder V8.0 (https://rshine.einsteinmed.edu/). No association between KFERQ‐like motif‐containing proteins and discordant gene regulation was observed (Figure S13), providing no evidence that alteration of chaperone mediated autophagy is responsible for the discordance between mRNA and protein expression.

### Assessment of the Impact of miRs in the Regulation of Discordant Gene Expression

A list of predicted gene targets for the shear‐regulated miRs was generated by combining miRDB v6.016, TarBase v9.017, MirTarBase v9.018, and TargetScan v8.0 databases. Subsequent analysis of enrichment of these predicted gene targets with the discordantly regulated genes failed to find any association (Figure S14), suggesting that potential miR‐gene interaction alone is insufficient to identify any targeting of the discordant gene expression and that other factors influence their interaction.

**Figure 7:**
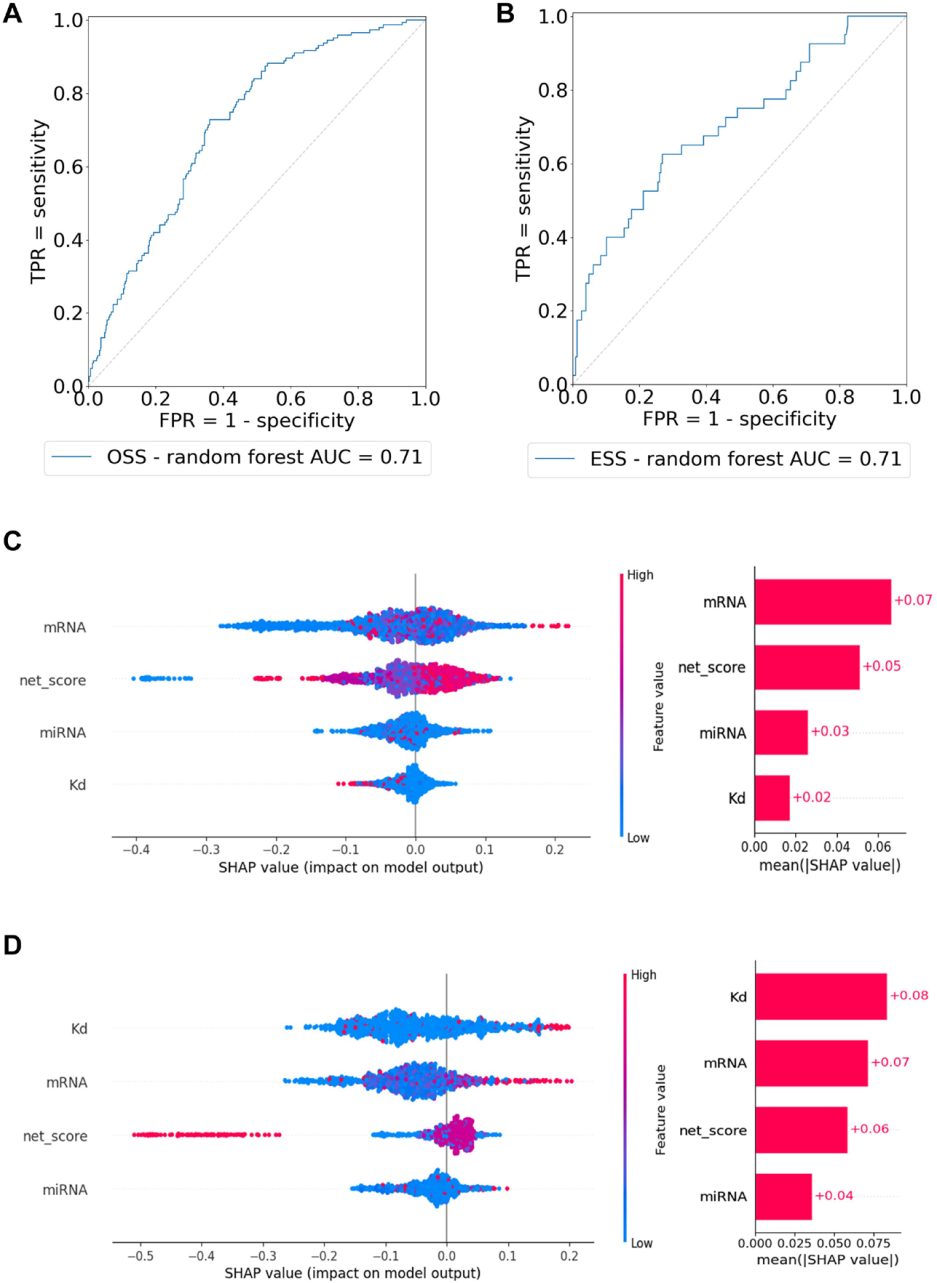
Machine learning models to investigate the impact of shear‐regulated miRNAs on discordant gene regulation. **A** and **B**. ROC curve on the OSS and ESS samples. The plots show the performances of the random forest models on the test set. The *x* axis shows the false positive rate given by subtracting all the specificity values from 1. The *y* axis shows the true positive rate calculated from the sensitivity. **A**, Performance of the random forest classifier on the samples exposed to OSS. **B**, Performance of the random forest classifier on the samples exposed to ESS. **C** and **D**, SHAP values beeswarm plots and bar plots. On the right, the beeswarm plots display an information‐dense summary of how the top features in a data set affect the model's output. A single dot on each feature row represents each SHAP value related to a feature. The SHAP value of that feature determines the x position of the dot, and dots “pile up” along each feature row to show density. Color is used to display the original value of a feature. **C**, SHAP value distribution of each value of the random forest trained on the OSS samples. **D**, SHAP value distribution of each value of the random forest trained on the ESS samples. ESS indicates elevated shear stress; miRNA, microRNA; OSS, oscillatory shear stress; ROC, receiver operating characteristic; and SHAP, Shapley Additive Explanations.

Exploratory analysis using multiple computational models identified that a random forest classifier was able to identify an interaction between shear regulated miRs and discordantly regulated gene expression, with area under the curve scores of 0.71, for both OSS and ESS subsets of genes (Figure 7A and 7B). This required the inclusion of additional data, including the relative read counts of target mRNAs, the number of shear regulated miRs that were predicted to target an individual gene, the total read counts of the targeting miRs and the calculated binding energy of the miR‐mRNA interaction. Shapley Additive Explanations values were calculated and visualized to interpret feature contributions to the prediction of the random forest model, highlighting that the mRNA read count (equivalent to the concentration) had the greatest contribution to the classifier (Figure 7C and 7D). Although difficult to predict on an individual miR–gene interaction, this analysis does support an important role for shear‐regulated miRs in controlling the expression of genes where there is discordance between the mRNA and protein abundance.

## DISCUSSION

Endothelial cells under normal physiological flow adopt a quiescent, low proliferative phenotype in vivo that could be considered the basal state of an endothelial cell. By contrast, endothelial cells in regions of disturbed flow are primed for inflammation and have higher levels of proliferation, senescence and endothelial to mesenchymal transition.^5^, ^22^, ^23^, ^24^ By contrast, the impact of elevated flow, which is experienced on the upstream surface of stenotic plaques where most plaque ruptures or erosions occur, has received less attention to date.^9^, ^12^, ^21^

The ability to run multiple parallel flow chambers at once has allowed a full suite of RNA and proteomic analyses to be performed on samples collected at the same time from the same passage of each donor HCAEC, offering a unique opportunity to directly compare the effects of different flow environments on HCAEC gene expression. This has highlighted several shear‐regulated pathways that have received little scrutiny in previous analyses. Both RNA and proteomic analyses highlighted the increase in cholesterol biosynthesis under LSS and amplified in ESS, which may denote a requirement for enhanced membrane rigidity to withstand higher frictional forces or potentially to “desensitize” mechanosensitive cell surface receptors upon achieving a homeostatic response by increasing membrane stiffness. The reciprocal decrease in cholesterol biosynthesis under OSS may promote membrane fluidity, allowing greater sensitivity of mechanosensors. The necessity for synthesis of cholesterol rather than simple uptake and use from low‐density lipoprotein is unclear and may point to intermediate metabolites playing an important role in endothelial function, implicated by the pleiotropic effects of statins on endothelial cells.^25^, ^26^ In addition, recent identification of cholesterol‐related genetic variants with impact on endothelial function were identified.^27^ The resultant polygenic risk score highlighted individuals who derived greater benefit from aggressive statin treatment, again stressing the importance of this axis in regulating endothelial function and resultant impact on CAD.

*SMAD1* upregulation under oscillatory flow increases the capacity for TGFβ/BMP signaling. Similarly, a decrease in CD109 mRNA and protein expression under oscillatory flow would be predicted to promote TGFβ signaling through reducing TFGβ receptor internalization.^28^ Both observations are consistent with disturbed flow providing a more permissive environment of endothelial‐to‐mesenchymal transition to occur.^24^, ^29^, ^30^ Mild alteration of splicing observed for both *ACVRL1* (OSS) and *ACVR1* (ESS) have the capacity to provide an additional layer of shear modulation of this important pathway that regulates EC function.^31^, ^32^, ^33^

Other notable changes in protein expression included several regulators of coagulation, with downregulation of *TFPI* (tissue factor pathway inhibitor) and urokinase‐type plasminogen activator *PLAU* under ESS, which may promote a more coagulation‐permissive environment. *ADAMTS1* mRNA and protein downregulation under OSS may potentiate VEGF (vascular endothelial growth factor) signaling and angiogenic potential of ECs in this flow environment. Lastly, *MFGE8* (Medin precursor protein) was upregulated under ESS.

In addition to overall regulation of mRNA expression, both OSS and to a greater extent ESS, caused a shift in transcript isoform selection, extending previous descriptions of flow‐induced changes in RNA splicing.^18^, ^34^ Strikingly, our analysis identified novel shear‐regulated RNA isoforms in human endothelial cells. Of potential relevance to plaque erosion, altered splicing of *FYN*, *NRF1*, *IRF7*, *IRAK4*, *IRF3*, and *STING1* may modify the cell stress response under elevated flow. Similarly, differential splicing of *PXN* and *ITGB4* has the capacity to modify adhesome function. These require further analysis to define the impact on cellular processes relevant for plaque erosion.

Several components of the YAP/TAZ‐TEAD (Hippo) signaling pathway also showed alternative transcript selection under shear stress. The exclusion of the Sorbin Homology domain of *SORBS3* under OSS, combined with notable expansion of the disordered domain of *PDLIM5* may provide additional regulation for the observed increase of YAP/TAZ signaling under disturbed flow^35^, ^36^ shown to increase the development of atherosclerosis in hyperlipidemic models.^37^, ^38^ The implication of the observed regulation of *TEAD1* and *PARD3* under ESS on this pathway are as yet undetermined.

Our analysis highlights significant enrichment of both OSS and ESS DEGs at genetic loci associated with CAD risk. Of the 375 linkage disequilibrium‐filtered single nucleotide variants with MAF > 0.005 from 2 recent CAD multipopulation meta‐analyses,^10^, ^11^ 36% of the CAD‐associated variants had at least 1 OSS DEG within 250 kb, and 26.9% were within 250 kb of at least 1 of the ESS DEGs. Overall, 246 (65%) loci had at least 1 OSS or ESS DEG within the same linkage disequilibrium block and within 250 kb of the lead SNV. The mechanism through which these CAD‐associated variants mediate their effects on the pathogenesis of atherosclerosis is largely unknown; however, this analysis provides a rationale for further study in HCAECs to determine any regulation of the known atheroprotective effects of laminar flow or plaque progression in regions exposed to elevated flow.

Using multiple omic analyses from parallel samples collected at the same time has allowed an exploratory analysis of the impact of shear‐regulated miRs to be assessed. The best model performance was obtained using a random forest classifier and required the inclusion of mRNA read counts, the number of shear regulated miRs that were predicted to target an individual gene, the total read counts of the targeting miRs and the calculated binding energy of the miR‐mRNA interaction to perform well, suggesting these features are functionally important in determining whether gene expression is affected by miRs. Indeed, one of the biggest contributions to the model was the relative abundance of the target gene mRNA and miR, features that are not currently considered within online target prediction tools. The area under the curve score of 0.71 suggests that shear‐regulated miRs do play a role in the expression of genes whose mRNA and protein levels are discordantly regulated by shear stress. Although our classifier achieved good performance, incorporating additional relevant features in the future could further enhance its accuracy and further improvements of the model are needed for a priori predictions whether an individual miR‐mRNA interaction will affect gene expression.

The results presented here have relevance for potential priming of signaling pathways implicated in plaque erosion. Most plaque erosions occur on the upstream surface of the plaque and through the area of maximal stenosis, where the endothelium is chronically exposed to elevated flow.^6^, ^7^, ^8^, ^39^, ^40^, ^41^ We have previously shown that elevated flow amplifies the stress response to simulated smoking that is mediated through Nrf2 (encoded by *NFE2L2*). The resulting shear‐dependent upregulation of *OSGIN1* and *2* coordinated a combined Nrf2, *HSF1*, and *IRF*‐like response, resulting in disrupted proteostasis and loss of adhesion.^21^ Data presented here highlight an enhanced IFN (gamma)‐like transcriptional response (Figure 1D) under elevated flow potentially contributing to an erosion‐permissive gene expression pattern under elevated flow. In addition, elevated flow upregulation of *PLA2G3* (Figure 1C) and *PLA2G4C*, with *PTGS2* and *PTGDS* upregulated by LSS and ESS compared with OSS (Data Set S1), provides greater capacity to generate 15dPGJ2, a known activator of both Nrf2 and *HSF1*,^42^, ^43^ again potentially providing an erosion‐permissive priming of the cell stress response, while inhibiting inflammasome activation.^44^ Analysis of the whole cell proteome revealed an increase in the hyaluronan receptor CD44 under elevated flow. The underlying matrix of plaques that erode are rich in hyaluronan,^40^, ^45^, ^46^, ^47^ priming endothelial inflammation through activation of both *TLR4* and *CD44*. Both downstream signaling pathways converge, with *TLR4‐MYD88‐IRF* activation being augmented by *CD44‐ERK‐AKT* signaling, which also increases *IRF3* activation,^48^, ^49^ synergizing with *OSGIN*‐coordinated cell‐stress signaling.^21^ Concomitantly, elevated flow also increases the expression of *IRF7* (Data Set S1), increasing potential for IRF‐signaling. Therefore, the data presented here add further detail to support the hypothesis that elevated flow modifies endothelial phenotype, creating a permissive phenotype that amplifies signaling pathways implicated in promoting plaque erosion.

A smaller proportion of erosions occur in regions exposed to disturbed flow (^41^ analyzed in more detail^6^). Of potential relevance, the pathway relating to pathogen induced cytokine storm signaling was consistently enhanced in HCAECs cultured under OSS at both the mRNA and protein level. In addition to multiple cytokines (Data Set S2) this pathway includes *GSDMD*, which is upregulated in OSS and is implicated in cigarette tar induced pyroptosis in endothelial cells as a potential driver of plaque erosion.^50^ Concomitant with this, the upregulation of *STING1* and regulation of IRF splicing under OSS provides an integrated signaling paradigm where cigarette smoke and other cell stressors may promote plaque erosion under elevated flow through Nrf2‐driven upregulation of *OSGIN1* and *OSGIN2*,^21^ and in regions exposed to OSS cigarette tar and mitochondrial dysfunction would promote pyroptosis,^50^

This current study has a number of limitations. First, the study was performed with HCAECs from 3 independent male donors. A lack of high‐quality HCAECs from female donors has hampered the analysis of sex‐specific shear regulation of gene expression. Completion of a sex‐specific analysis is an immediate aim as it may highlight differently regulated pathways of relevance to plaque erosion. Both the mechanism of regulation and the biological significance of alternative splicing are yet to be elucidated. Significant additional investigation will be necessary to assess the impact of this largely unexplored component of gene regulation in endothelial function and pathophysiology. Although a number of OSS and ESS DEGs were identified to locate within 250 kb and reside within the same linkage disequilibrium block as SNVs associated with CAD risk, further analysis is required to identify if the shear‐regulated DEGs represent the causal gene for the association with CAD. The sensitivity of detection of proteins expression by liquid chromatography tandem mass spectrometry is lower for the proteomic analysis compared with the RNAseq analysis, highlighted by an observable relationship between fold change and *P* value (Figure 3B through 3D). This limited the identification of genes with discordant mRNA‐protein regulation to the genes where the proteins were significantly detected by liquid chromatography tandem mass spectrometry.

## CONCLUSIONS

In conclusion, the present study extends many previous investigations showing that the flow environment regulates HCAEC gene expression by providing a unique integrated data set of linked mRNA, miR, and proteomic data from 3 donors under oscillatory, normal physiological, and elevated flow. The data presented here clearly demonstrate the uniqueness of the response of HCAEC to elevated flow at every level of gene expression control and highlight changes in gene expression that may contribute to a phenotypic shift that is permissive for plaque erosion.

## Sources of Funding

The work was supported by British Heart Foundation [grant: PG/17/67/33218]. DGM is supported by the Leicester British Heart Foundation Research Excellence Award (RE/24/130031) and the van Geest Foundation Heart and Cardiovascular Diseases Research Fund and was awarded a British Heart Foundation Accelerator Early Careers Researcher Interdisciplinary Fellowship and pump‐priming funding from the Leicester British Heart Foundation Accelerator Award (AA/18/3/34220).

## Disclosures

None.

## Supporting information

Data S1. Supplemental Methods
Tables S1–S2
Figures S1–S14
Data Sets S1–S3
