# Supplementary material for: Multiomic Reference Map of Endothelial Mechanosensitive Pathways Under Athero‐ and Erosion‐Prone Flow
Source: J Am Heart Assoc. 2026 Jun 23;15(13):e048290. doi: 10.1161/JAHA.125.048290 (PMC13477354; doi:10.1161/JAHA.125.048290)
Supplement: Supplementary file 1 [file JAH3-15-e048290-s001.zip › jah370634-sup-0001-Supinfo01..pdf]

SUPPLEMENTAL MATERIAL

Data S1. Supplemental Methods

HCAEC culture and generation of RNA and protein samples for analysis

Confluent monolayers of three independent batches of male HCAECs purchased from Promocell were exposed to oscillatory ±0.5Pa 1Hz (OSS), normal physiological laminar shear stress (1.5Pa LSS) and elevated laminar shear stress (7.5Pa ESS) for 72 hours using our established parallel plate flow chambers as previously described[9 12 13 14]. In more detail, HCAECs were expanded to passage 3-4 to provide sufficient cells to seed all of the required slides for both protein and RNA analysis to be performed under all shear conditions at the same time. Plain glass slides (checked for thickness to be 1mm ±0.05mm) were prepared by autoclaving in deionised water, before being drained and re-autoclaved, to ensure any chemicals that can leach out of the slides are removed during the first autoclave run. The sterile slides were then coated with gelatin (Attachment Factor, Gibco) for >24 hours at 4°C within an adapted silicone gasket prepared by cutting out the intervening segments from 8-chamber FlexiPerm silicon insert (Order number 94.6032.039, inset image, total surface area ~9.4cm^2). This ensures that the subsequent cell seeding and culture is constrained within the area of the slide that experiences uniform flow within the parallel plate flow apparatus, away from each end and the sides of the flow chamber. HCAECs were seeded within the silicon gasket seeded at moderately high density (2.5x10^5 cells per gasket) and cultured for 72 hours to ensure confluence and allow stable cell-cell junctions to form and extracellular matrix deposition to occur. Subsequently the slides were assembled within parallel plate flow apparatus[9], and cultured under flow for 72 hours in Promocell media MV2 omitting the hydrocortisone from the media and supplementing the media with 3.35% dextran (Sigma 31390) to increase the viscosity to mirror that of plasma[9]. Following 72 hours of culture under flow, cells were lysed for either RNA or protein purification. The ability to run >25 flow chambers at a time allowed the protein and RNA samples for each individual donor to be prepared in a single experiment, allowing direct comparison across the different omic analyses for each HCAEC donor. Each donor RNA or protein sample represents one replicate in each analysis, therefore there was n=3 RNA or protein samples analysed.

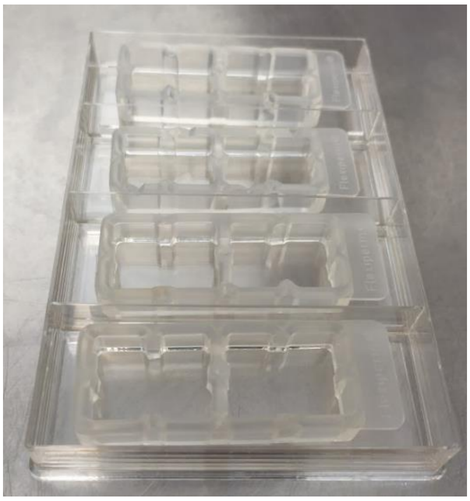

RNAseq, miRseq data generation and analysis

TruSeq Stranded RNA 76 bp paired-end sequencing was performed on Illumina HiSeq4000. Sequencing quality was assessed using FastQC (v 0.11.3), FastQ Screen (v 0.9.2), trimmed with Trimmomatic (v 0.36) and mapped against the reference genome (hg38) using STAR (version 2.7.2b). Counts per gene were calculated using STAR annotation from GENCODE 32. Normalisation and differential gene expression tests were performed in DESeq2 (v1.42.1) with confirmation of normalisation through p-value analysis. Transcripts with low counts were removed using independent filtering within DESeq2. The mRNAs with low counts across all samples were automatically excluded by setting the independent Filtering parameter to TRUE.

MicroRNA (miR) library preparation was performed using NEBNext® Multiplex Small RNA Library Prep Set and 76 bp paired-end sequenced performed on Illumina HiSeq4000. The R1 single-end RNA-seq reads were quality assessed using FastQC (v 0.11.3), FastQ Screen (v 0.9.2), quality trimmed and filtered for length with Cutadapt v1.18 (min length 18nt, max length 26nt), and mapped against the reference genome (hg38) using Bowtie (v 1.1.1). Counts per gene were calculated using feature Counts using annotation from miRBasev22. Normalisation, principal component analysis (PCA) and differential gene expression tests were performed in DESeq2 v1.20.0. Among the altered miRNAs, only those with at least 15 reads across all the samples were included within the analysis.

Pathway prediction based on mRNA changes was performed using Qiagen Ingenuity Pathway Analysis (IPA), within the Ingenuity platform (QIAGEN Inc., Redwood City, CA). Similarly, analysis of Upstream Regulators, Causal Networks, Diseases and Bio Functions and Regulator Effects were also generated within the Ingenuity platform.

Alternative splicing analyses

Differential alternative splicing analyses were performed comparing either oscillatory shear stress (OSS) or elevated shear stress (ESS) to laminar shear stress (LSS) using rMATS v.4.1.2[15]. Alternative splicing events were detected from BAM files aligned by hisat2 v.2.2.1[16] to the GRCh38 human genome reference (Ensembl v.112) and sorted by coordinates using samtools v.1.5[17]. rMATS analyses were carried out using the paired statistical model with a cutoff (--cstat) of 0.05. Results calculated by counting both the junction and the exon reads (JCEC) were

filtered to only include differential splicing events with a p-value ≤ 0.01 and predicted differential PSI (dPSI) of 8%.

Gene Ontology analyses of genes displaying these alternative splicing events were performed using GOstats v.2.60.0[18] to identified enriched biological processes (GOBP). Enrichment was calculated against genes with an average of at least 5 transcripts per million (TPM) across samples (universe), and p-values were adjusted by hypergeometric test. Redundant terms were filtered using Revigo v.1.8.1[19] with SimRel semantic similarity measure to only include GOBP with a dispensability score lower than 0.3 and containing between 5 and 500 genes.

All plots were produced in R v.4.1.2 using ggplot2 v3.5.1 and eulerr 7.0.2[20]. Snapshots from the Integrative Genomics Viewer (IGV) browser were generated using representative BAM files merged from the three batches of HCAECs with samtools v.1.5.

Identification of Differentially-Expressed Genes Within and Near To CAD-Associated Loci

CAD risk-associated variants were identified from two recent multi-population meta-analyses[10, 11]. Variants were filtered by LD using LDlink's SNPclip function using the European population and a R^2 threshold of 0.7. Where LD filtering would be applied to multiple variants, the variant with the lowest p-value in the two studies was selected as the lead variant. Variants not within the LDlink database were included in the final list of lead variants. The gene start site for the differentially-expressed genes was identified using Ensembl's BioMart function and genes with start sites within 250kb of the CAD-associated variants were identified. Circos plots of differentially-expressed genes near to CAD risk variants were plotted using the Python package pyCirclize (https://pypi.org/project/pyCirclize/). Where necessary to ensure legibility, gene labels were grouped together and the direction of the differential-expression is indicated by arrows for each gene.

A permutation test was performed to identify whether there was significant enrichment of CAD variants within 250kb of the start sites of differentially expressed genes. For each of the 100,000 permutations, a random variant was selected from well imputed variants (r^2>0.3) on the same chromosome for each of the 375 CAD variants (MAF>0.5%) and the number of differentially expressed genes within 250kb of each random variant was counted and the total found. Significance was estimated based on the number of times a permutation total exceeded the observed total.

Proteomic analysis

Sample preparation and analysis were performed as previously described[21]. In brief, protein samples (500 µg) obtained in SDS lysis buffer (2% SDS, 50 mM Tris pH 6.8, 10% glycerol) from different flow conditions were sonicated (300 seconds, 30W, Covaris LE220-PLUS), reduced (15 mM DTT, 56 °C, 45 minutes) and alkylated (50 mM iodoacetamide, room temperature, 45 minutes). Proteins were precipitated (4x volume acetone, -20 °C, 16 hours), collected by centrifugation (16,000xg 4 °C, 20 minutes) and resuspended in 0.1% (w/v) Rapigest (Waters) in 100 mM ammonium bicarbonate. Proteins were digested with trypsin at a 1:50 (w/v) ratio (37 °C, 16 hours with shaking at 250 rpm) and then acidified with 1% (v/v) trifluoracetic acid (TFA; 37°C ,45 minutes). Samples were desalted using Oasis HLB cartridges (Waters) according to manufacturer's instructions, eluting in binding solution: 80 % (v/v) acetonitrile (ACN), 5 % (v/v) TFA, 1M glycolic acid and stored at -20 °C.

For mass spectrometry, peptides were resuspended in 5% (v/v) ACN / 1% (v/v) formic acid and analysed by liquid chromatography (LC) tandem mass spectrometry (LC-MS/MS) using an UltiMate 3000 Rapid Separation LC (RSLC, Dionex Corporation) coupled to a Q Exactive HF (Thermo Fisher Scientific) mass spectrometer. Peptides were separated using a 75 mm x 250 µm i.d. 1.7 µM CSH C18, analytical column (Waters) with the LC gradient from 95% buffer A (0.1% (v/v) FA in water) and 5% buffer B (0.1% (v/v) FA in acetonitrile) to 7% buffer B at 1 minute, 18% buffer B at 58 minutes, 27% buffer B in 72 minutes and 60% buffer B at 74 minutes at 300 nL min-1. Peptides were selected for fragmentation automatically by data dependant analysis.

Relative protein abundances were calculated based on ion intensity as implemented in Proteome Discoverer (v2.5; ThermoFisher Scientific) [4], utilising the Protein FDR validator node set at high confidence equating to a 1% FDR cut off. RAW files were searched using SEQUEST-HT against the SwissProt and TREMBL human databases (release-2018_01). Carbamidomethylation of cysteine was set as a fixed modification; serine, threonine and tyrosine phosphorylation and oxidation of methionine were allowed as a variable modification. Mass tolerances for precursor and fragment ions were 10 ppm Da and 0.02 Da, respectively. Abundance ratios were calculated between treatment groups, together with adjusted P-values using the Benjamini-Hochberg method, in Proteome Discoverer.

Merging of the MiRNA databases

The miRNA-mRNA interactions were obtained merging 4 public miRNA-mRNA interaction databases (miRDB v6.0[22], TarBase v9.0[23], MirTarBase v9.0[24] and TargetScan v8.0[25]), utilising target genes identified by at least two databases. Nomenclature was aligned to miRBase Release 22[26].

Machine learning analysis of miR impact on discordant gene/protein expression

We divided miRNA-targeted genes into two classes, "class 0", containing genes whose behaviour is consistent between the transcriptomics and proteomics levels, and "class 1", where any changes were discordant. More precisely, class 0 comprises (a) genes with no significant changes at either level (mRNA adj p-value > 0.05 and protein adj p-value > 0.05), and (b) genes with changes at both levels in the same direction (mRNA adj p-value < 0.05 and protein adj p-value < 0.05 and mRNAs log2FC > 0 and protein log2FC > 0). Class 1 comprises (a) genes with changes only at the proteomics level (mRNA adj p-value > 0.05 and protein adj p-value ≤ 0.05), (b) genes with changes only at the transcriptomics level (mRNA adj p-value ≤ 0.05 and protein adj p-value > 0.05), and (c) genes with changes at both levels but in opposite directions (mRNA adj p-value < 0.05 and protein adj p-value ≤ 0.05 and mRNAs log2FC > 0 and protein log2FC < 0 or vice versa).

Classes 0 and 1 were defined separately for each biological contrast (OSS vs LSS or ESS vs LSS). Our goal is to develop ML models to predict classes 0 and 1.

- Model features

Four model features were used for the classification. These features are: concentration of the mRNAs and miRNAs (both measured as RPKM counts), net-effect score (calculated as the difference between up-regulated miRNAs and down-regulated miRNAs targeting the same mRNA) and constant of dissociation of each miRNA-mRNA interaction (calculated using Vienna RNA[27] using the sequences of the miRNAs of the 3'UTR regions of the mRNAs identified using APAtrap[28])

- Preparation of the dataset

A dataset containing the binary variable and all the model features was prepared for each condition to perform the binary classification. To consider the variability among donors, each gene in each sample was treated as separate entry (individual gene data points). The dataset

was then spitted into train set (80%) and test set (20%) using test_train_split function from scikit-learn v1.5[29]. After splitting the data, the train and test set were standardised separately using RobustScaler function from scikit-learn v1.5.

- Training and testing of the model

To optimize model performance, a Random Forest classifier was trained with a 10-fold cross validation and hyperparameter tuning using the RandomForestClassifier and GridSearchCV functions from scikit-learn v1.5. The model was evaluated using the ROC curves, focusing on the AUC score for the overall performance of the model and on the sensitivity to understand the ability of the model to classify class 1 entries.

- Interpretation of the classification

The SHAP values were calculated and visualised using the SHAP library[30] to interpret the contribution of each feature to the model's predictions.

Table S1. Principle Component Analysis (PCA) plot of samples following DESeq2 analysis and mean read counts of arterial, vein and capillary markers from ref 15.

	Normalised read count (Mean, n=3)		
Gene	OSS	LSS	ESS
SEMA3G	2097	833	1396
NOTCH4	2806	1309	1558
GJA5	832	328	915
GJA4	1579	479	14
ACKR1	28	78	48
VCAM1	63	62	30
CA4	21	16	0
RGCC	1874	1052	1468
HEY1	418	526	271
F2RL3	612	2198	1947

Table S2. Top 5 up- and down-regulated genes in each discordantly regulated gene test group for both oscillatory (OSS) and elevated shear stress (ESS) conditions**.**

OSS vs LSS - Test group 1

	Gene Symbol	mRNA log2FC	mRNA adjusted p-value	protein log2FC	protein adjusted p-value
Top 5 down-regulated mRNAs	VAMP5	-0,14	0,79	-5,97	0
	EXOC1	0,32	0,35	-5,97	0
	ACP5	-1,97	0,55	-5,57	0
	EBP	-1,07	0,13	-5,32	0
	TRIM4	-0,1	0,72	-5,16	0

	Gene Symbol	mRNA log2FC	mRNA adjusted p-value	protein log2FC	protein adjusted p-value
Top 5 up-regulated mRNAs	TGFBI	-0,4	0,61	4,09	0
	MAPKAPK2	-0,16	0,43	4,26	0
	RAB27A	0,42	0,15	4,6	0
	TAGLN	-0,07	0,98	5,03	0
	DHRS4	-0,21	0,65	5,78	0

ESS vs LSS - Test group 1

	Gene Symbol	mRNA log2FC	mRNA adjusted p-value	protein log2FC	protein adjusted p-value
Top 5 down-regulated mRNAs	SLCO2A1	-0,92	0,67	-5,11	0
	USP39	0,14	0,51	-4,68	0
	APLN	-0,68	0,67	-4,21	0
	XRCC1	-0,31	0,46	-4,18	0
	PLVAP	-0,85	0,34	-4,13	0

	Gene Symbol	mRNA log2FC	mRNA adjusted p-value	protein log2FC	protein adjusted p-value
Top 5 up-regulated mRNAs	TTYH3	-0,08	0,85	3,05	0
	NAGLU	-0,6	0,3	4,18	0
	RAB27A	-0,45	0,14	4,97	0
	EDIL3	0,33	0,62	5,04	0
	CLEC16A	0,39	0,11	6,57	0

OSS vs LSS - Test group 2

	Gene Symbol	mRNA log2FC	mRNA adjusted p-value	protein log2FC	protein adjusted p-value
Top 5 down-regulated mRNAs	LTF	-4,52	0	0,63	0,22
	SULF1	-4,5	0	-1,02	0,16
	ITGB4	-3,55	0,01	-0,92	0,31
	ASS1	-3,3	0	0,4	0,53
	HIST2H2BE	-2,86	0	-0,7	0,39

	Gene Symbol	mRNA log2FC	mRNA adjusted p-value	protein log2FC	protein adjusted p-value
Top 5 up-regulated mRNAs	AK5	3,7	0	-0,77	0,53
	TFPI2	3,06	0,05	0,41	0,53
	KRT7	2,72	0	0,25	0,72
	GMFB	2,52	0	0,28	0,75
	FABP5	2,49	0	-0,85	0,25

ESS vs LSS - Test group 2

	Gene Symbol	mRNA log2FC	mRNA adjusted p-value	protein log2FC	protein adjusted p-value
Top 5 down-regulated mRNAs	TFPI2	-3,64	0,03	-0,04	0,96
	KRT7	-2,61	0	-0,31	0,9
	APOM	-2,55	0,04	0,46	0,52
	PTX3	-2,49	0	-1,36	0,25
	BGN	-2,21	0	-1,2	0,17

	Gene Symbol	mRNA log2FC	mRNA adjusted p-value	protein log2FC	protein adjusted p-value
Top 5 up-regulated mRNAs	HIST1H4H	2,52	0,01	-0,62	0,44
	ALDOC	2,59	0	0,17	0,74
	HIST2H2BE	2,9	0	-0,95	0,07
	HIST1H4E	3,47	0	-0,62	0,44
	PI16	4,04	0,02	-0,4	0,95

OSS vs LSS - Test group 3

	Gene Symbol	mRNA log2FC	mRNA adjusted p-value	protein log2FC	protein adjusted p-value
Top 5 down-regulated mRNAs	CLEC3B	-5,41	0,04	1,31	0,01
	SERPINE2	-2,23	0	3,29	0
	COL1A1	-2,22	0,01	3,49	0
	ADAMTSL1	-1,19	0,01	1,3	0,05
	STIM2	-1,05	0	2,06	0

	Gene Symbol	mRNA log2FC	mRNA adjusted p-value	protein log2FC	protein adjusted p-value
Top 5 up-regulated mRNAs	GBA2	1,32	0	-1,95	0,01
	CAVIN2	1,32	0,01	-2,35	0
	CLU	1,42	0	-1,45	0,02
	VWF	1,59	0,04	-2,27	0
	FABP4	4,8	0	-3,57	0

ESS vs LSS - Test group 3

	Gene Symbol	mRNA log2FC	mRNA adjusted p-value	protein log2FC	protein adjusted p-value
Top 5 down-regulated mRNAs	FBLN2	-4,21	0,02	4,01	0
	COL1A1	-2,53	0,01	2,06	0
	EDNRB	-2,05	0	1,65	0,01
	TGFBI	-1,57	0,01	2,03	0
	SULT1B1	-1,27	0,02	1,19	0,02

	Gene Symbol	mRNA log2FC	mRNA adjusted p-value	protein log2FC	protein adjusted p-value
Top 2 up-regulated mRNAs	GALNS	0,69	0	-2,68	0
	GNG5	1,2	0,01	-2,76	0

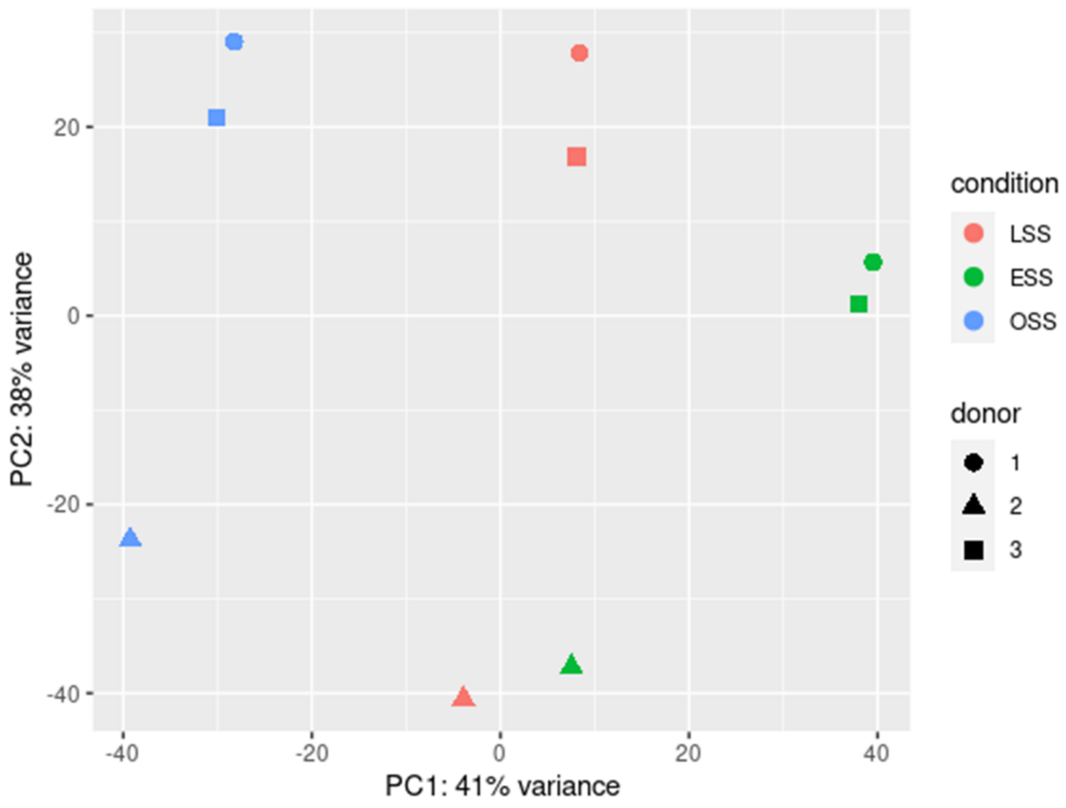

Figure S1. Principle Component Analysis (PCA) plot of samples following DESeq2 analysis and mean read counts of arterial, vein and capillary markers from ref 15.

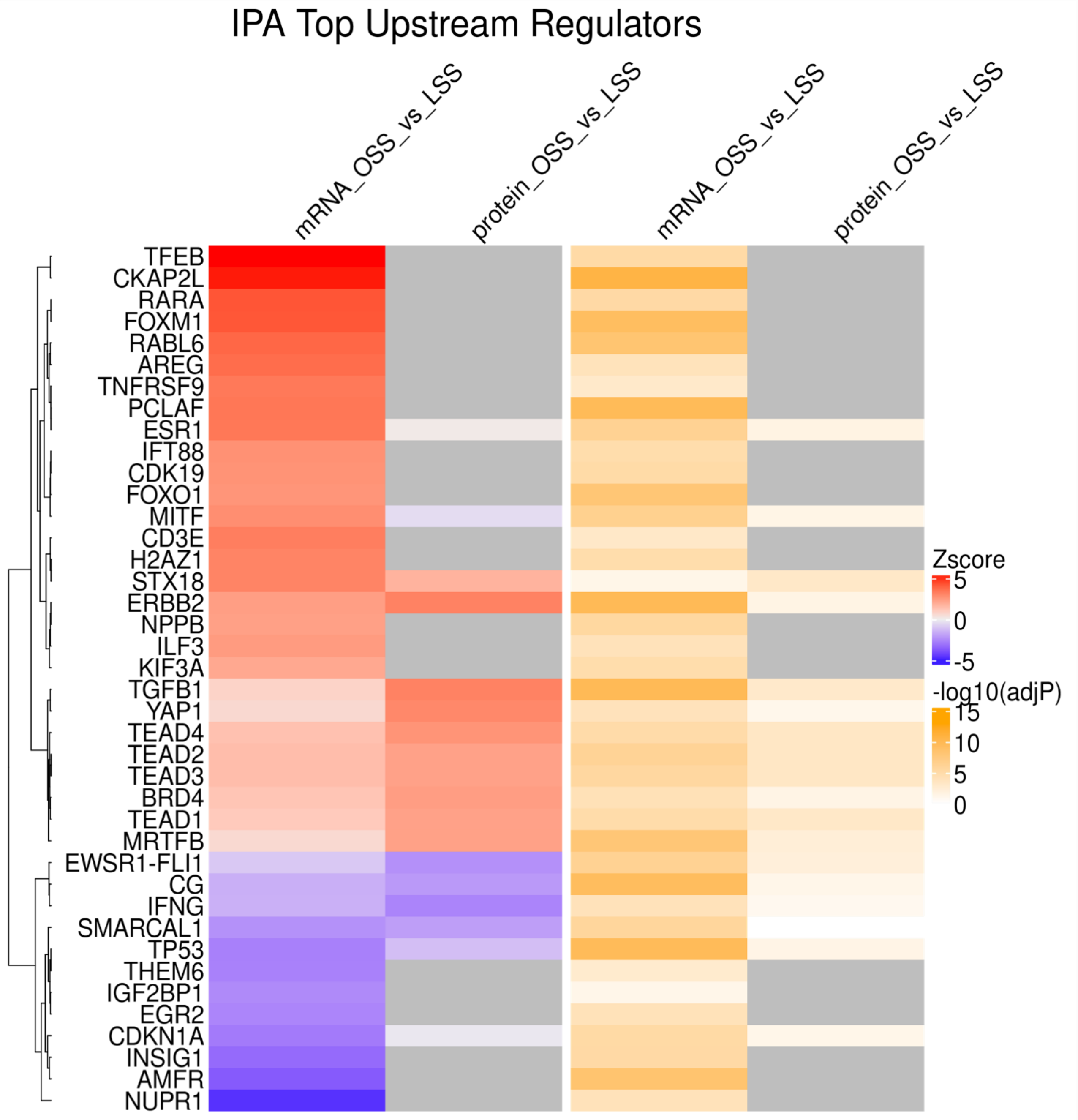

Figure S2. Top IPA predicted regulators for genes modulated by oscillatory shear stress (OSS) compared to physiological laminar shear stress (LSS).

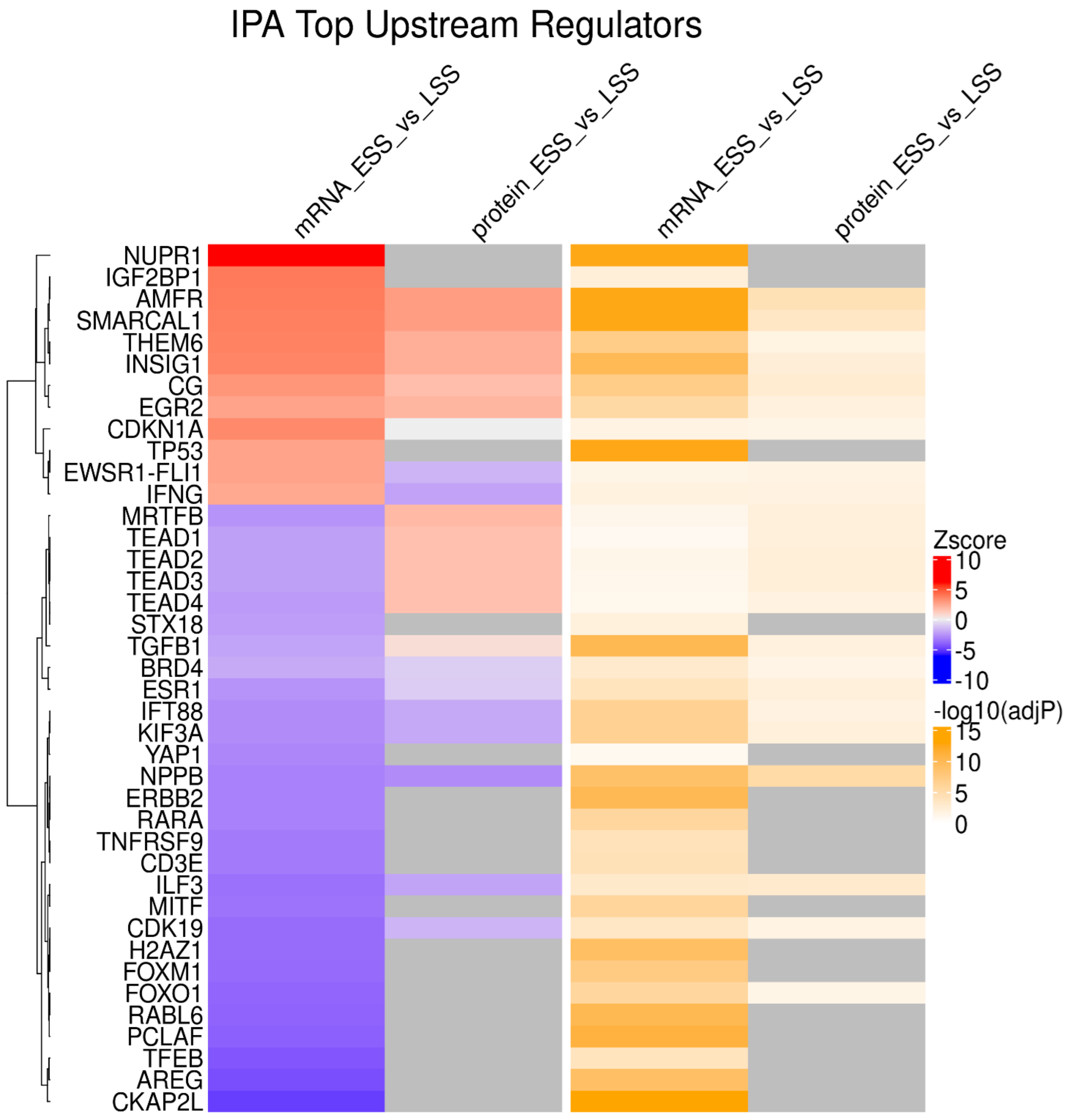

Figure S3. Top IPA predicted regulators for genes modulated by elevated shear stress (ESS) compared to physiological laminar shear stress (LSS).

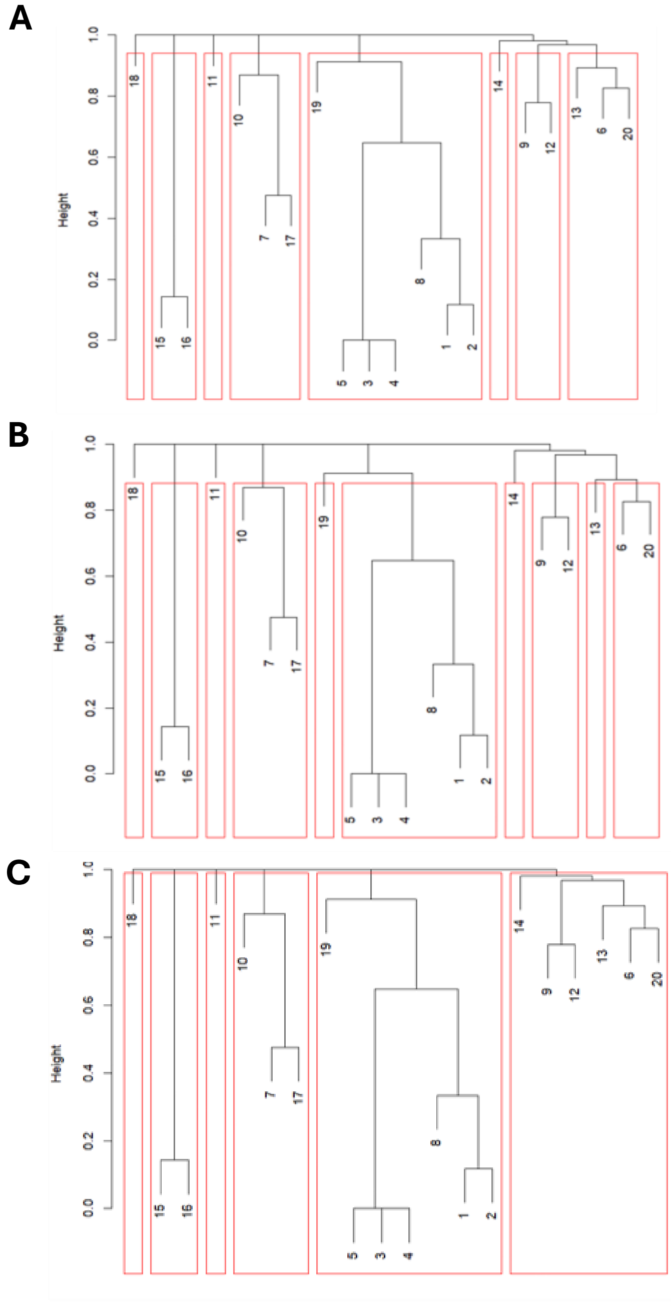

ID	PATHWAY NAME
1	Cholesterol biosynthesis
2	Superpathway of Cholesterol Biosynthesis
3	Cholesterol Biosynthesis I
4	Cholesterol Biosynthesis II (via 24,25-dihydrolanosterol)
5	Cholesterol Biosynthesis III (via Desmosterol)
6	Pathogen Induced Cytokine Storm Signaling Pathway
7	Kinetochore Metaphase Signaling Pathway
8	Activation of gene expression by SREBF (SREBP)
9	Tumor Microenvironment Pathway
10	Mitotic Roles of Polo-Like Kinase
11	Keratinization
12	Pulmonary Fibrosis Idiopathic Signaling Pathway
13	Class A/1 (Rhodopsin-like receptors)
14	Airway Pathology in Chronic Obstructive Pulmonary Disease
15	Mevalonate Pathway I
16	Superpathway of Geranylgeranyldiphosphate Biosynthesis I (via Mevalonate)
17	Mitotic Prometaphase
18	Potassium Channels
19	LXR/RXR Activation
20	Agranulocyte Adhesion and Diapedesis

Figure S4 Dendrograms of pathway clustering on the oscillatory (OSS) vs physiological laminar shear stress (LSS) – generated in R using binary distance between the gene sets associated with each pathway, and hierarchical clustering via the hclust algorithm. Red rectangles indicate the division into: **(A)** 6 clusters, **(B)** 8 clusters and **(C)** 10 clusters.

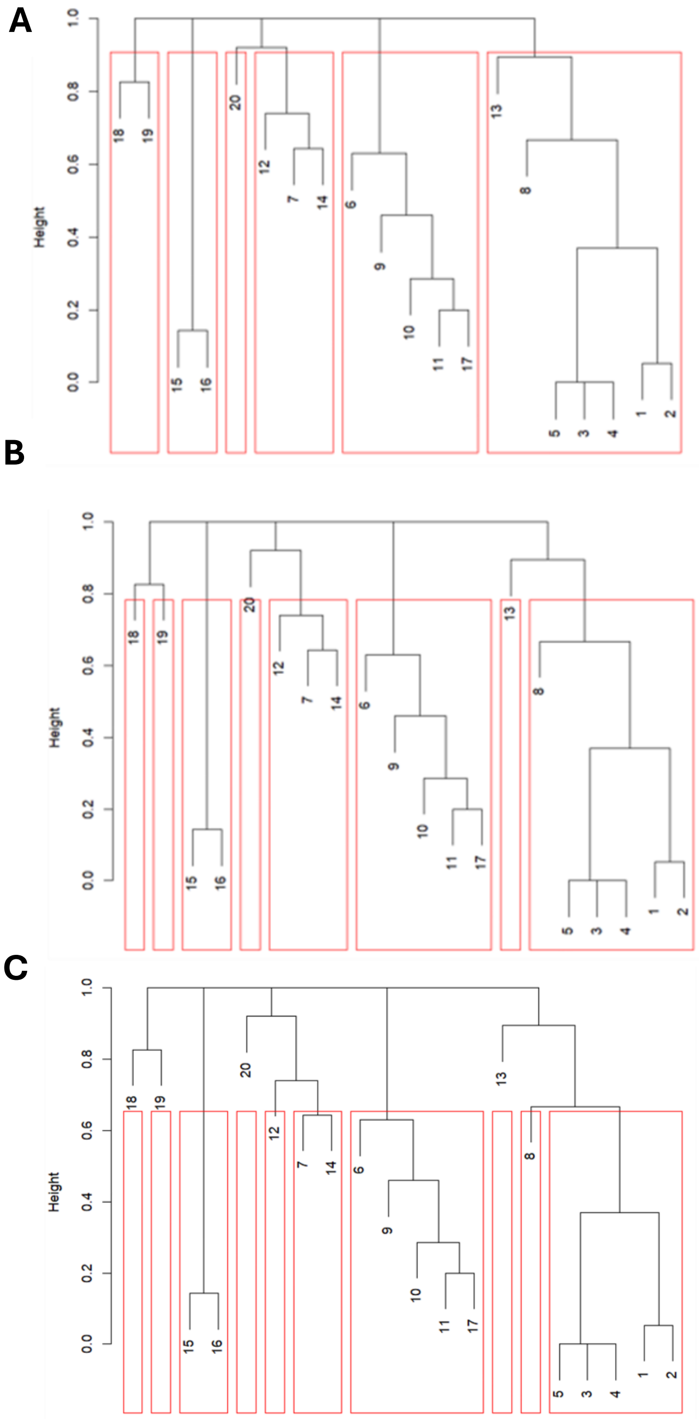

ID	PATHWAY NAME
1	Cholesterol biosynthesis
2	Superpathway of Cholesterol Biosynthesis
3	Cholesterol Biosynthesis I
4	Cholesterol Biosynthesis II (via 24,25-dihydrolanosterol)
5	Cholesterol Biosynthesis III (via Desmosterol)
6	Pathogen Induced Cytokine Storm Signaling Pathway
7	Kinetochore Metaphase Signaling Pathway
8	Activation of gene expression by SREBF (SREBP)
9	Tumor Microenvironment Pathway
10	Mitotic Roles of Polo-Like Kinase
11	Keratinization
12	Pulmonary Fibrosis Idiopathic Signaling Pathway
13	Class A/1 (Rhodopsin-like receptors)
14	Airway Pathology in Chronic Obstructive Pulmonary Disease
15	Mevalonate Pathway I
16	Superpathway of Geranylgeranyldiphosphate Biosynthesis I (via Mevalonate)
17	Mitotic Prometaphase
18	Potassium Channels
19	LXR/RXR Activation
20	Agranulocyte Adhesion and Diapedesis

Figure S5 Dendrograms of pathway clustering on the elevated (ESS) vs physiological laminar shear stress (LSS) – generated in R using binary distance between the gene sets associated with each pathway, and hierarchical clustering via the hclust algorithm. Red rectangles indicate the division into: **(A)** 6 clusters, **(B)** 8 clusters and **(C)** 10 clusters.

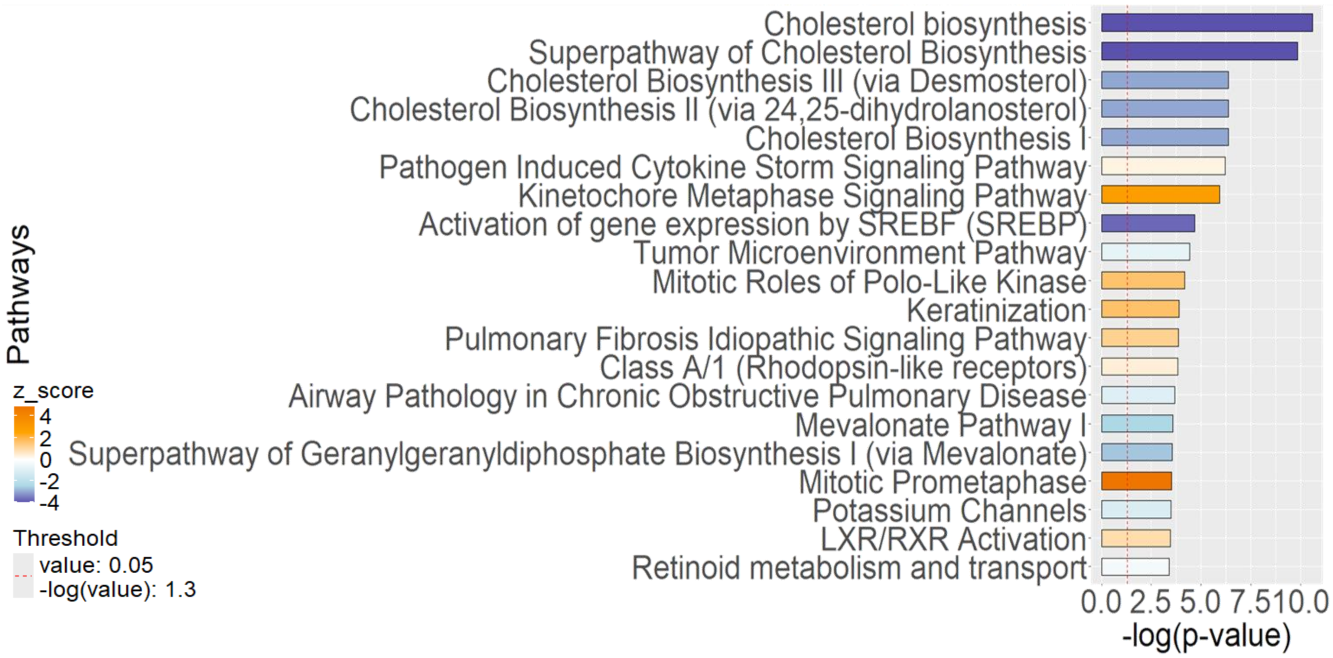

Figure S6. Uncondensed pathway analysis of genes with altered expression in oscillatory flow. Generated by IPA

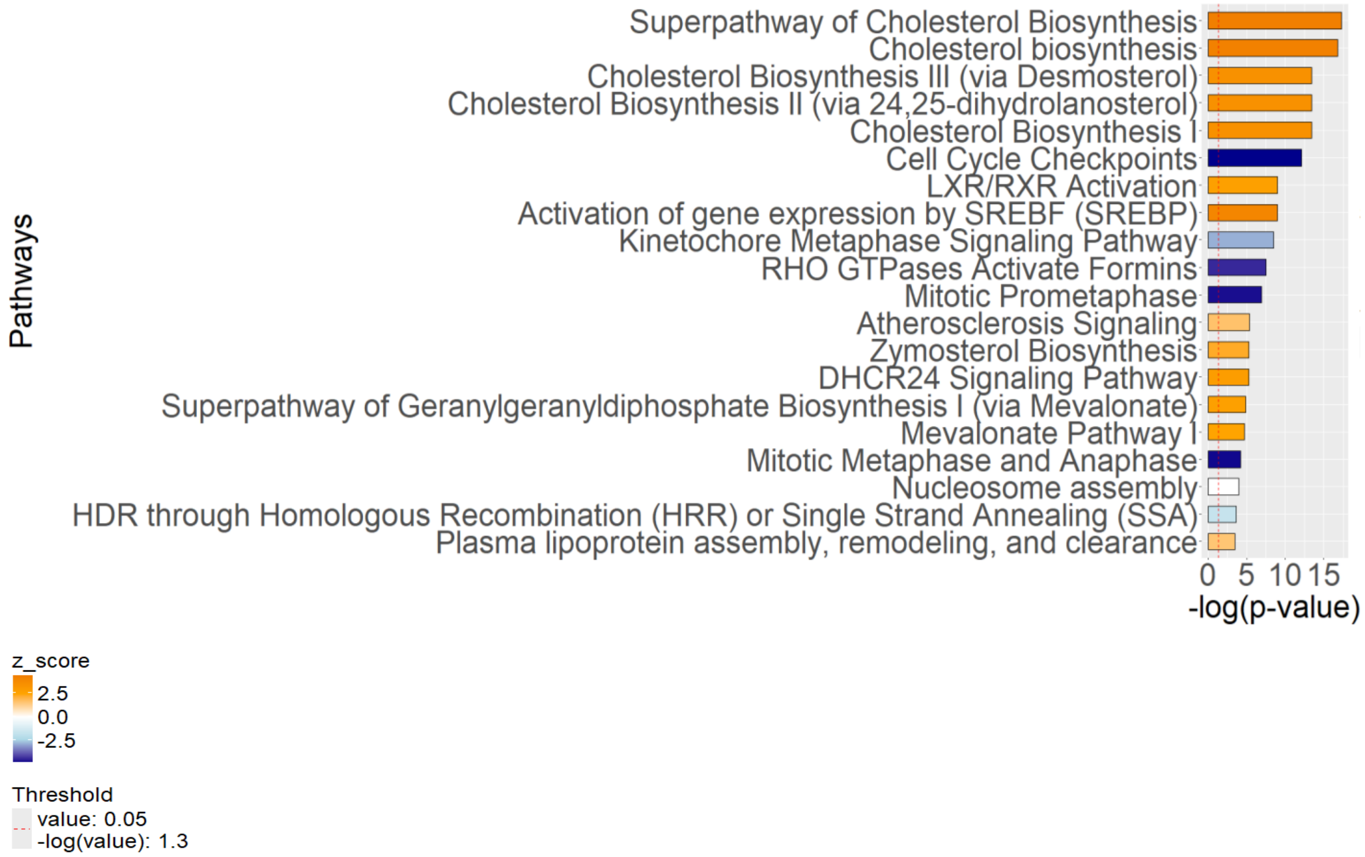

Figure S7. Uncondensed pathway analysis of genes with altered expression in elevated flow. Generated by IPA

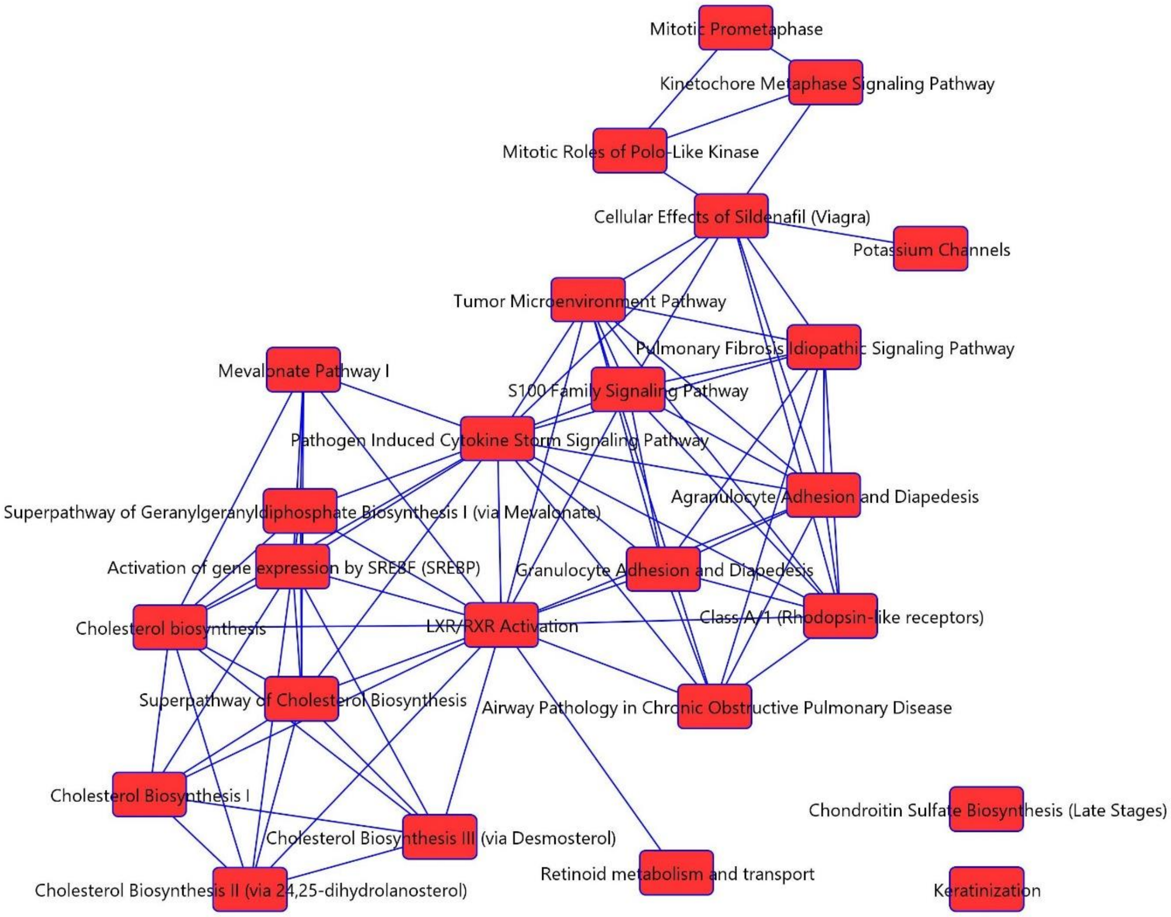

Figure S8. Networks of overlapping IPA canonical pathways, oscillatory shear stress.

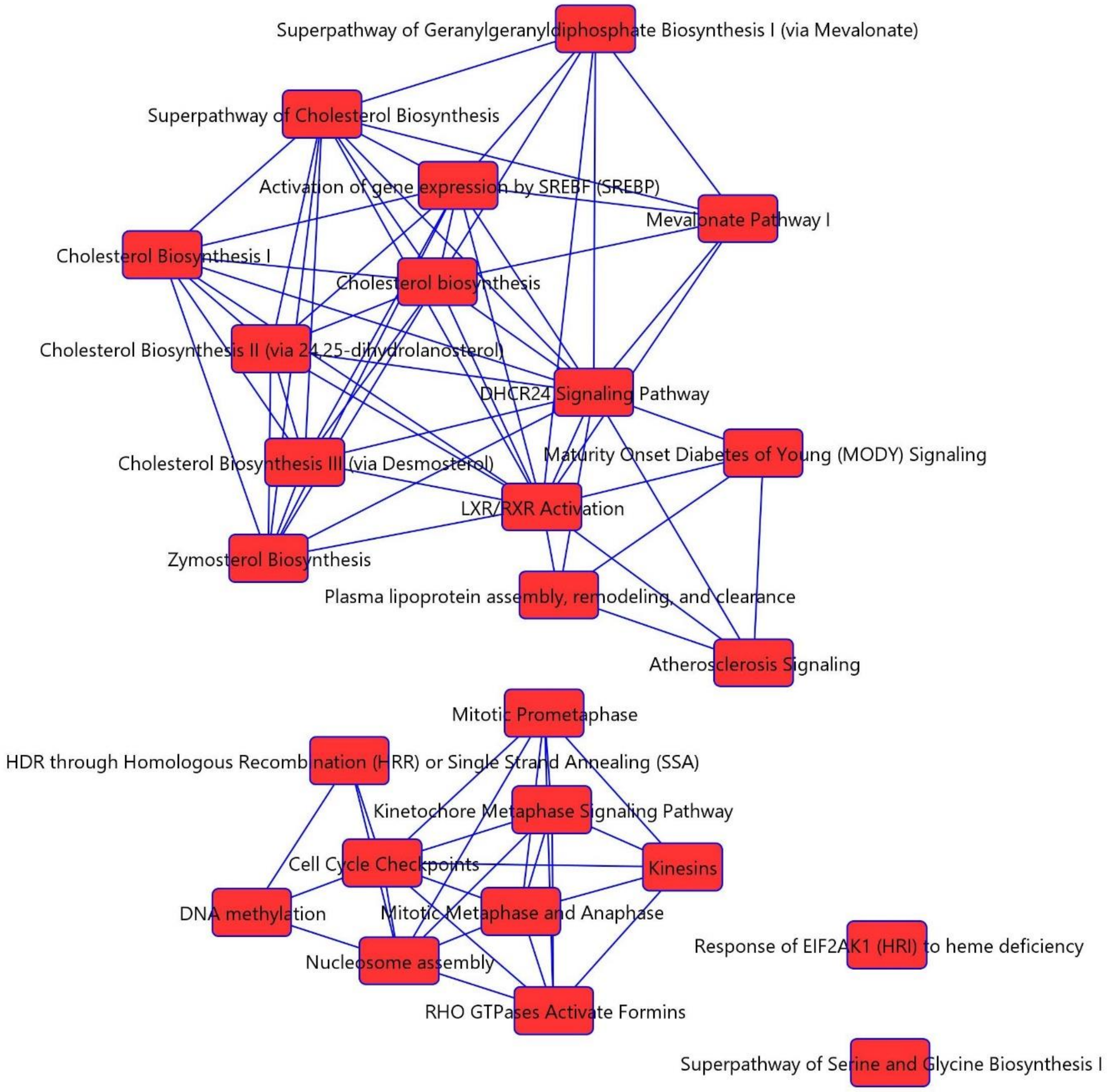

Figure S9. Networks of overlapping IPA canonical pathways, B: elevated shear stress.

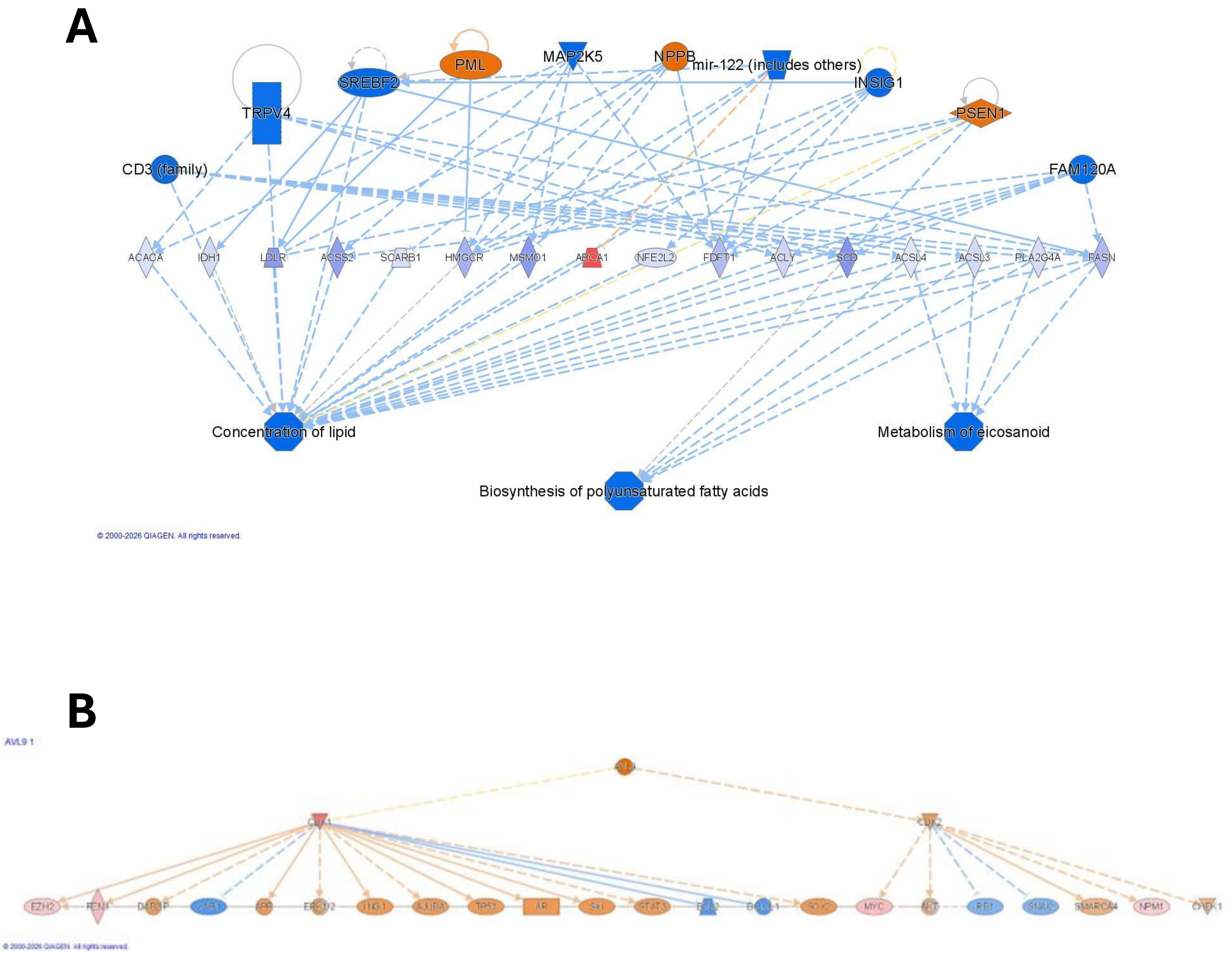

Figure S10. Predicted network analysis comparing oscillatory (OSS) v elevated shear stress (ESS), A) Regulator effects controlling lipid and cholesterol synthesis; B) Inter-related casual networks regulating changes in gene expression between OSS and ESS.

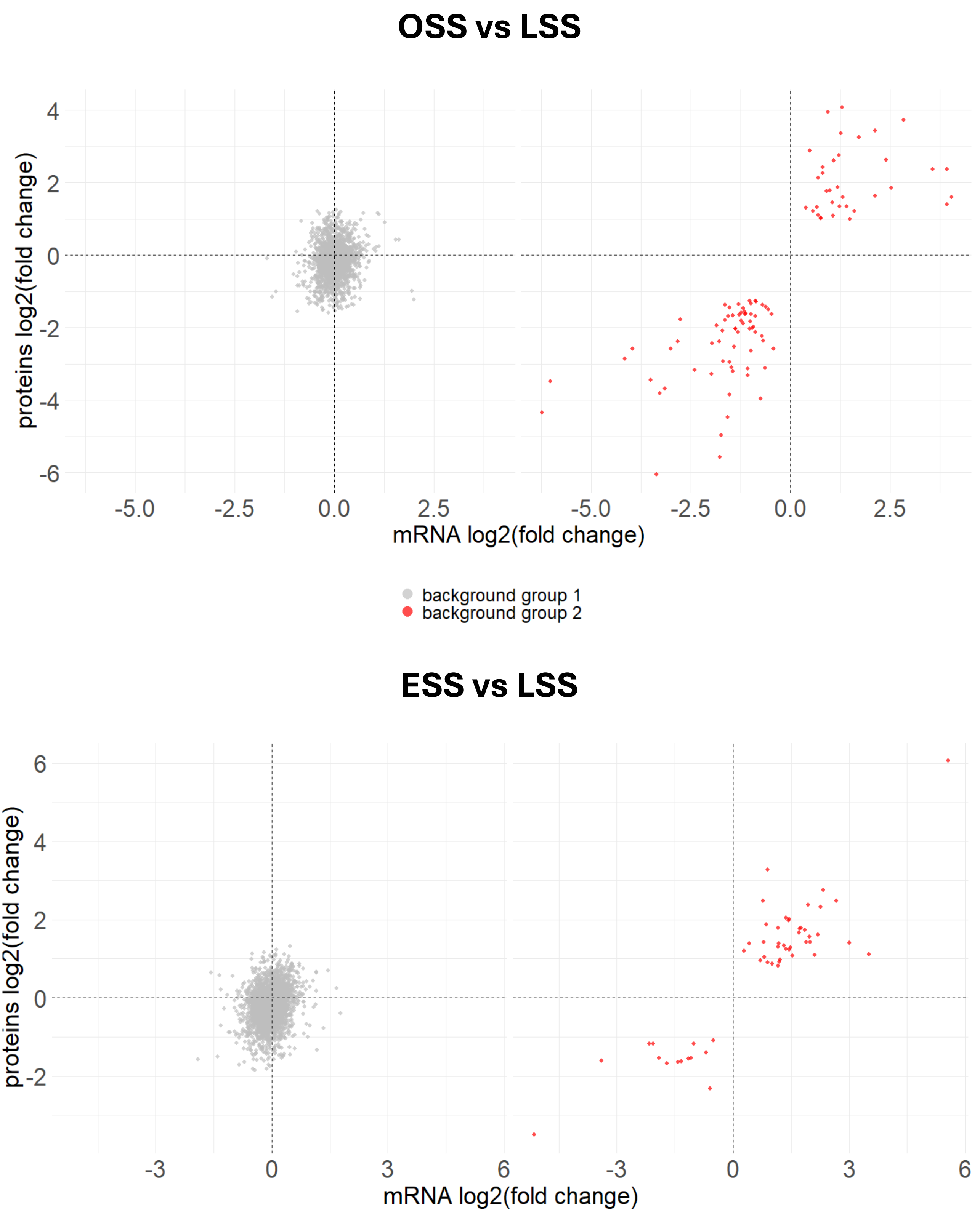

Figure S11. Scatter plots of the background groups. The plots show the relationship between the log2FC of the genes (x-axis) and the log2FC of the associated proteins (y-axis). Each gene and the respective protein are represented by a single dot. The colour of the dots represents background group 1 (grey) and background group 2 (red). At the top, genes in background group 1 and 2 are defined for oscillatory (OSS) vs physiological laminar shear stress (LSS). Lower figures, genes in background group 1 and 2 are defined for elevated shear stress (ESS) vs LSS.

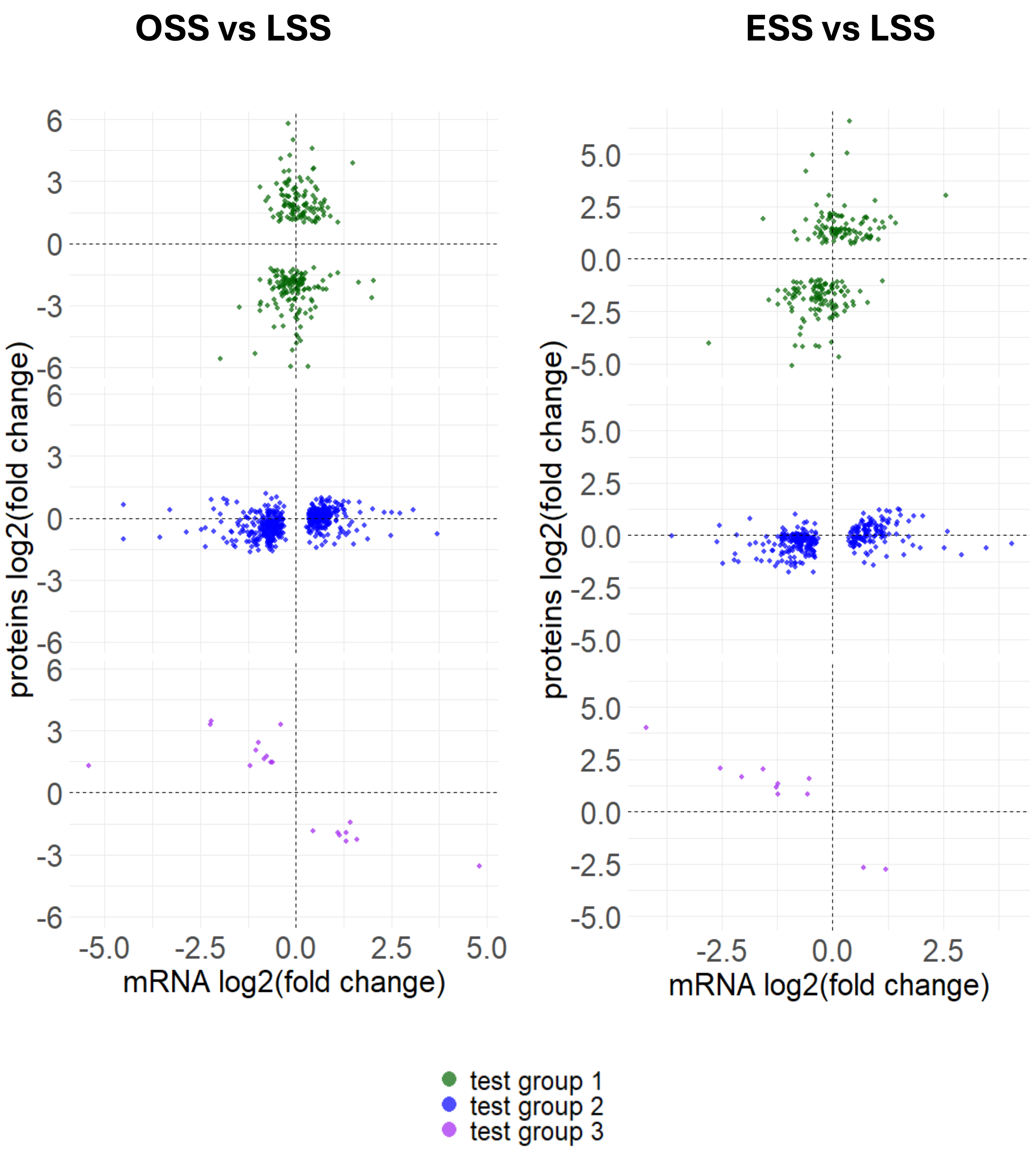

Figure S12. Scatter plots of the test groups. The plots show the relationship between the log2FC of the genes (x-axis) and the log2FC of the associated proteins (y-axis). Each gene and the respective protein are represented by a single dot. The color of the dots represents test group 1 (green), test group 2 (blue) and test group 3 (purple). On the left, genes in test group 1, 2 and 3 are defined based on oscillatory (OSS) vs physiological laminar shear stress (LSS) comparison. On the right, genes in test group 1, 2 and 3 are defined for elevated shear stress (ESS) vs LSS contrast.

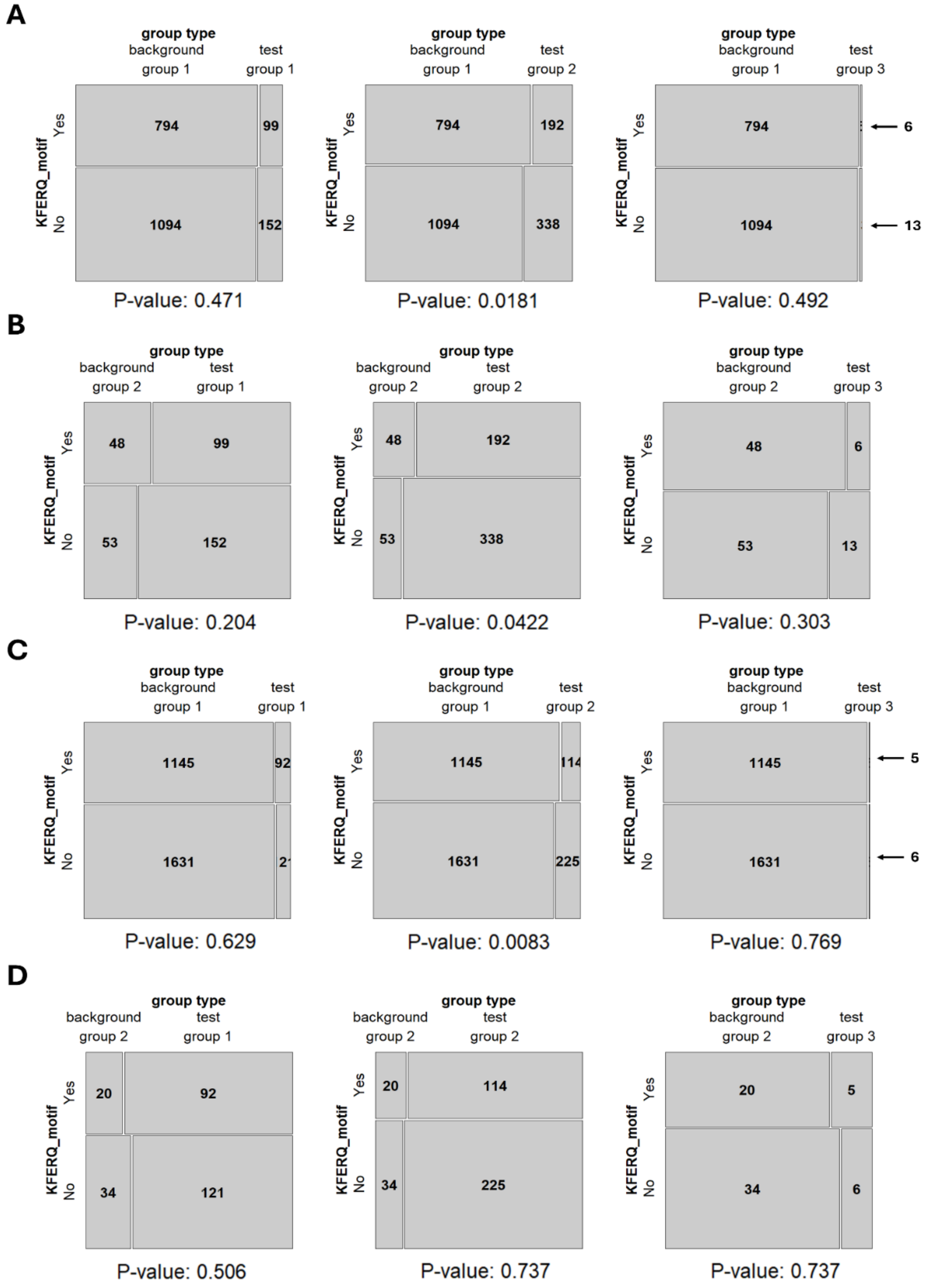

Figure S13. Tests of Association for KFERQ-like motifs

To investigate the relationship between miRNA impact and discordant changes in the previously defined test groups, we assessed associations using either Fisher's exact test or the Chi-square test, depending on the sample size. None of the tests were found to be significant, as all were associated with a p-value greater than 0.05. As a result, there was no evidence of a significant association between a gene belonging to one of the discordantly regulated expression groups and its predicted inclusion of a KFERQ motif

OSS vs LSS

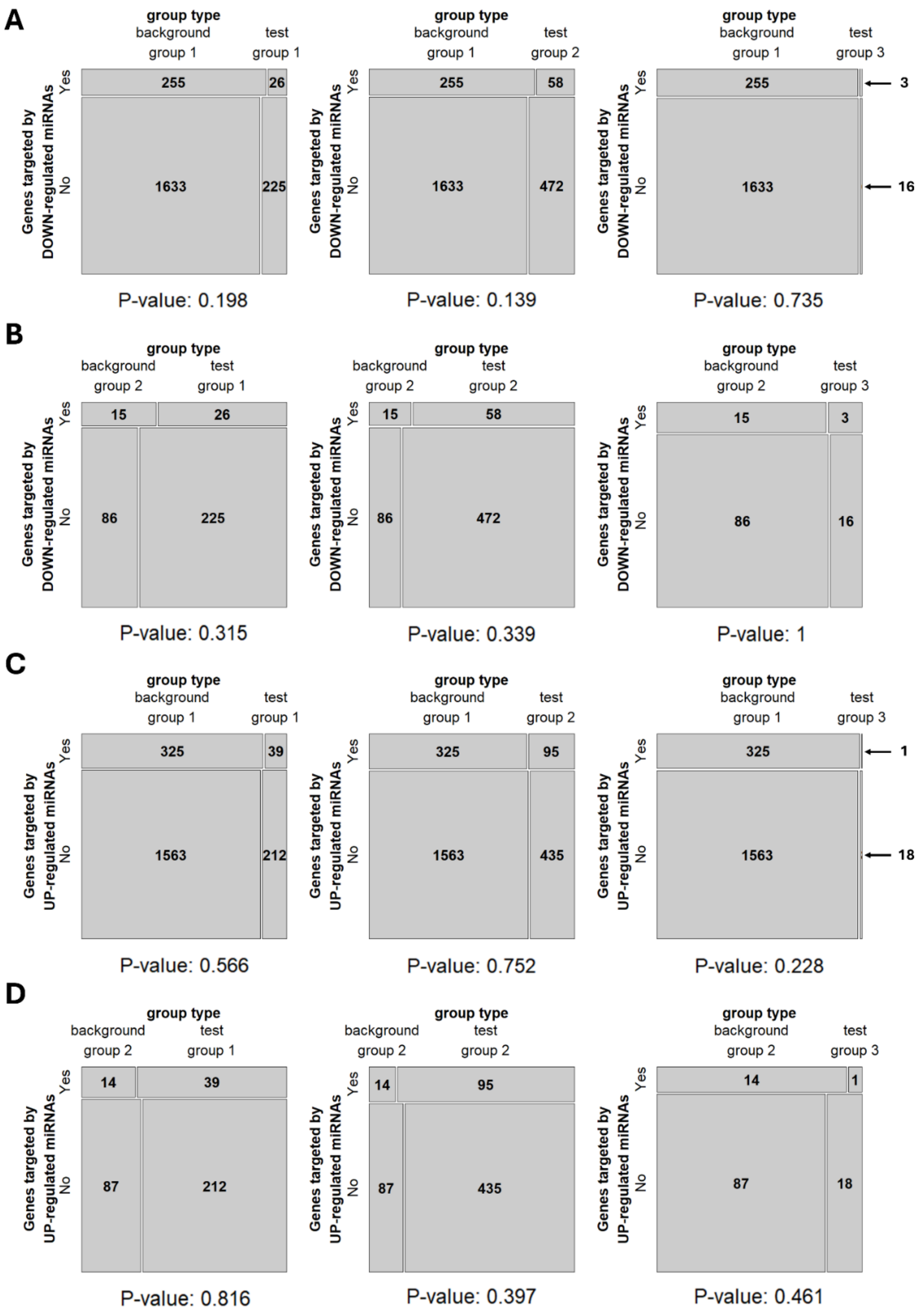
A
group type
background
group 1
test
group 1
Genes targeted by DOWN-regulated miRNAs
Yes
No
255
26
1633
225
P-value: 0.198
group 2
255
58
1633
472
P-value: 0.139
group 3
255
3
1633
16
P-value: 0.735
B
group 2
15
26
86
225
P-value: 0.315
15
58
86
472
P-value: 0.339
15
3
86
16
P-value: 1
C
Genes targeted by UP-regulated miRNAs
325
39
1563
212
P-value: 0.566
325
95
1563
435
P-value: 0.752
325
1
1563
18
P-value: 0.228
D
14
39
87
212
P-value: 0.816
14
95
87
435
P-value: 0.397
14
1
87
18
P-value: 0.461

ESS vs LSS

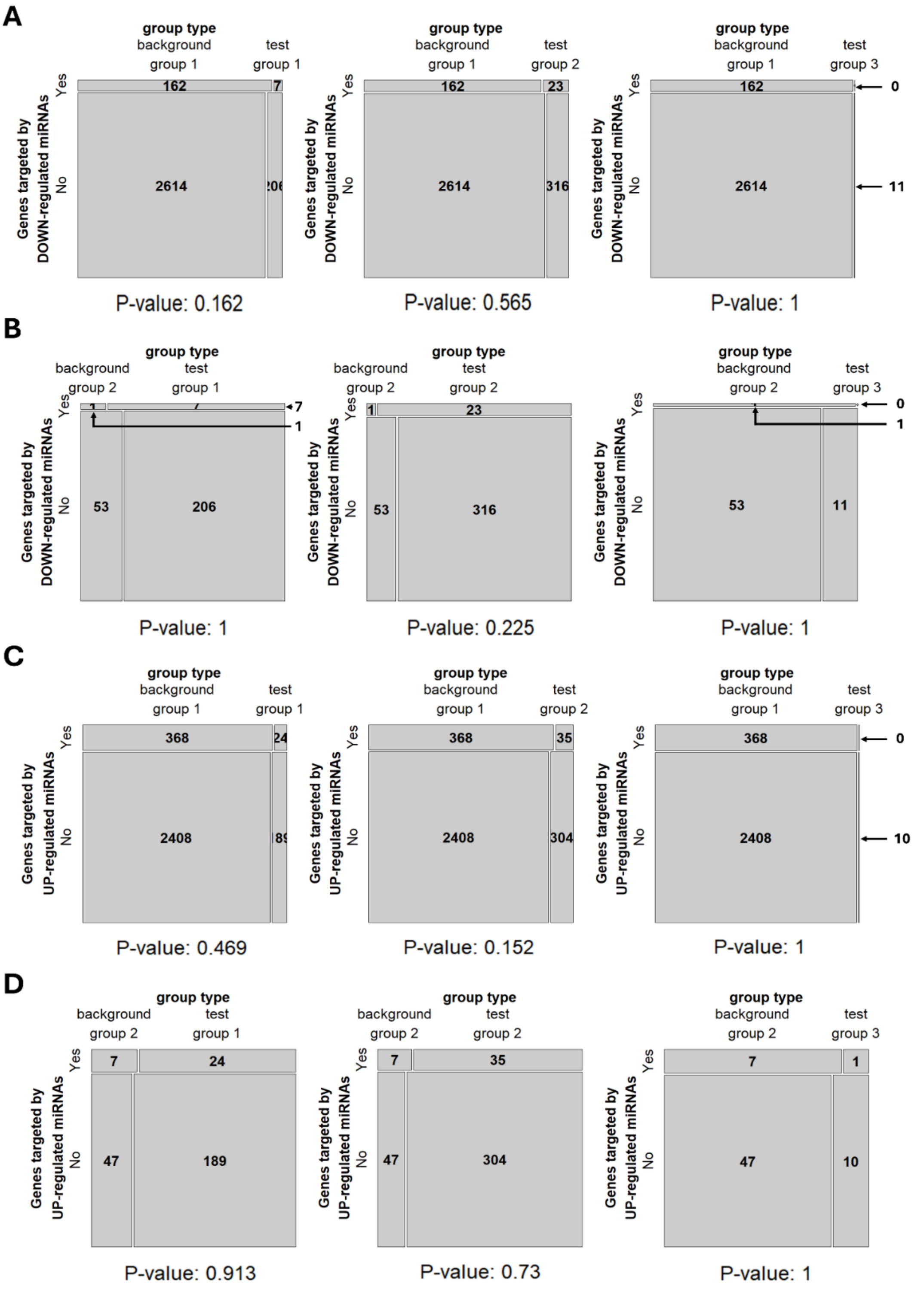

Figure S14. Tests of Association for predicted miR targets

To investigate the relationship between miRNA impact and discordant changes in the previously defined test groups, we assessed associations using either Fisher's exact test or the Chi-square test, depending on the sample size. None of the tests were found to be significant, as all were associated with a p-value greater than 0.05. As a result, there was no evidence of a significant association between a gene belonging to one of the discordantly regulated test groups and its predicted targeting by an up-regulated or down-regulated miRNA.

Supplemental Datasets – see Excel files

Dataset S1 – Excel data file with tables containing the full lists of genes and data

- Sheet 1OSS v LSS differentially regulated genes, |log2fc| >1, padj<0.05
- Sheet 2ESS v LSS differentially regulated genes, |log2fc| >1, padj<0.05
- Sheet 3OSS v ESS differentially regulated genes, |log2fc| >1, padj<0.05
- Sheet 4Genes predicted to be regulated by upstream regulator pathways OSS v LSS
- Sheet 5Genes predicted to be regulated by upstream regulator pathways ESS v LSS
- Sheet 6OSS v LSS hypervariable genes between donors |log2fc| >3, padj>0.05
- Sheet 7ESS v LSS hypervariable genes between donors |log2fc| >3, padj>0.05
- Sheet 8Splicing OSS
- Sheet 9Splicing ESS
- Sheet 10 Splicing OSS Gene ontology
- Sheet 11 Splicing ESS Gene ontology
- Sheet 12 OSS DEGs within 250kb of CAD-Associated Variants
- Sheet 13 ESS DEGs within 250kb of CAD-Associated Variants
- Sheet 14 OSS v LSS differentially regulated miR, |log2fc| >1, padj<0.05
- Sheet 15 ESS v LSS differentially regulated miR, |log2fc| >1, padj<0.05
- Sheet 16 OSS v ESS differentially regulated miR, |log2fc| >1, padj<0.05
- Sheet 17 OSS v LSS differentially regulated proteins, |log2fc| >1, padj<0.05
- Sheet 18 ESS v LSS differentially regulated proteins, |log2fc| >1, padj<0.05
- Sheet 19 OSS v ESS differentially regulated proteins, |log2fc| >1, padj<0.05
- Sheet 20 OSS v LSS discordant gene set 1
- Sheet 21 OSS v LSS discordant gene set 2
- Sheet 22 OSS v LSS discordant gene set 3
- Sheet 23 ESS v LSS discordant gene set 1
- Sheet 24 ESS v LSS discordant gene set 2
- Sheet 25 ESS v LSS discordant gene set 3
- Sheet 26 OSS v LSS concordant genes
- Sheet 27 ESS v LSS concordant genes

Dataset S2– Excel data file containing IPA generated canonical pathways, upstream regulators, causal networks, diseases or functions annotation for differentially regulated mRNAs.

Dataset S3– Excel data file containing IPA generated canonical pathways, upstream regulators, causal networks, diseases or functions annotation for differentially regulated proteins.
